# Supplementary material for: Evaluating the next generation of RSV intervention strategies: a mathematical modelling study and cost-effectiveness analysis
Source: BMC Med. 2020 Nov 18;18:348. doi: 10.1186/s12916-020-01802-8 (PMC7672821; doi:10.1186/s12916-020-01802-8)
Supplement: Supplementary file 1 — Additional file 1. Supplementary methods text. [file 12916_2020_1802_MOESM1_ESM.pdf]

Additional file 1 for Evaluating the next generation of RSV intervention strategies: A mathematical modelling study and cost-effectiveness analysis
Methods

by David Hodgson, Richard Pebody, Jasmina Panovska-Griffiths, Marc Baguelin, and Katherine Atkins

Contents

1 RSV model structure **2**
1.1 Maternal protection model 4
1.2 Contact matrices 5
1.3 Force of infection 7
1.4 Initial conditions 7
1.5 Model output 8

2 Parameterisation of prior distributions **9**
2.1 Duration of immunity 9
2.2 Duration of symptomatic infection 9
2.3 Susceptibility to infection 10
2.4 Asymptomatic infection 10
2.5 Transmission and initial parameters 10

3 Model-fitting **12**
3.1 Detection model 12
3.2 Calibration 14
3.3 Model choice 15
3.4 Posterior distributions 15
3.5 Implementation 15

4 Intervention model **17**
4.1 Palivizumab programme 17
4.2 Long-acting monoclonal antibodies programmes 20
4.3 Childhood/elderly vaccination programmes 23
4.4 Maternal vaccine programmes 26

5 Economic model **31**
5.1 Estimating annual incidence of outcomes 31
5.2 QALY loss due to death 32
5.3 Cost-effectiveness 32

1 RSV model structure

State	Description
$M(t)$	Number of individuals at time t who are completely protected from infection due to maternally-derived antibodies.
$S_i(t)$	Number of individuals at time t who are susceptible to acquiring an RSV infection, who have experienced i previous infections.
$E_i(t)$	Number of individuals at time t who are infected with RSV but are not yet infectious (i.e. exposed),who have experienced i previous infections.
$A_i(t)$	Number of individuals at time t who are infected with RSV, infectious and have no symptoms of RSV-related respiratory disease, who have experienced i previous infections (not including the current infection).
$I_i(t)$	Number of individuals at time t who are both infected with RSV, infectious and have symptoms of RSV-related respiratory illness, who have experienced i previous infections (not including the current infection).
$R_i(t)$	Number of individuals at time t who are completely protected from infection due to immunity acquired from natural-infection, who have experienced i infections (not including the one just experienced).
$Z(t)$	Cumulative number of new RSV infections at time t
$V_P(t)$	Number of individuals at time t who are completely protected from infection due to immunity acquired from administration of Palivizumab.
$V_M(t)$	Number of individuals at time t who are completely protected from infection due to immunity acquired from administration of long-acting monoclonal antibodies.

Table 1: Description of the epidemiological state variables of the RSV model, where $i \in \{0, 1, 2, 3\}$.

State	Description
η^a	Ageing rate from age group a to age group $a+1$.
$\mathfrak{p}^{a,b}$	Total number of daily physical contacts made by age group a with age group b.
$\mathfrak{c}^{a,b}$	Total number of daily conversational contacts made by age group a with age group b.
I_1	Initial proportion (at $t = 0$) of people who are infected (i.e. in epidemiological compartments E, I or A) with RSV (
I_2	Initial proportion (at $t = 0$) of people who not-infected but are protected (in epidemiological compartment R) from RSV

Table 2: Description of additional epidemiological parameters in the RSV model which are not included in **Table 1** of the main text.

In order to capture the heterogeneity in transmissive capacity across the population, we stratified the model into demographic groups according to their age (indicated by the superscript a). 25 age groups were considered, allowing for the dynamics of RSV incidence in infants to be closely monitored (age groups: <1, 1, 2, 3, 4, 5, 6, 7, 8, 9, 10, 11 months, and 1, 2, 3, 4, 5, 5–9, 10–14, 15–24, 25–34, 35–44, 45–54, 55-64, 65–74, 75+ years). The number of individuals, N^a, in each age group age, a, is calculated by multiplying the daily birth rate in 2018 for England and Wales, μ, by the number of days spent in each age group (d_a). Individuals in an epidemic compartment move to the next age group ($X^a \to X^{a+1}$) at rate $\eta^a = 1/(365\, d_a)$.[1]

The ODEs of the RSV transmission model for age group a are:

$$
\begin{aligned}
\dot{M^a} &= \overbrace{p_R\mu\mathbb{1}_1(a) - \xi M^a}^{\text{Transmission terms}} && \overbrace{-\eta^a M^a + \eta^{a-1}M^{a-1}}^{\text{Ageing terms}} \\
\dot{S_0^a} &= (1-p_R)\mu\mathbb{1}_1(a) + \xi M^a - \lambda_0^a(t)S_0^a && -\eta^a S_0^a + \eta^{a-1}S_0^{a-1} \\
\dot{E_0^a} &= \lambda_0^a(t)S_0^{a,s} - \sigma E_0^a && -\eta^a E_0^a + \eta^{a-1}E_0^{a-1} \\
\dot{A_0^a} &= p^a\sigma E_0^a - \gamma_0 A_0^a && -\eta^a A_0^a + \eta^{a-1}A_0^{a-1} \\
\dot{I_0^a} &= (1-p^a)\sigma E_0^a - \gamma_0 I_0^a && -\eta^a I_0^a + \eta^{a-1}I_0^{a-1} \\
\dot{R_0^a} &= \gamma_0 A_0^a + \gamma_0 I_0^a - \omega R_0^a && -\eta^a R_0^a + \eta^{a-1}R_0^{a-1} \\
\dot{S_1^a} &= \omega R_0^a - \lambda_1^a(t)S_1^a && -\eta^a S_1^a + \eta^{a-1}S_1^{a-1} \\
\dot{E_1^a} &= \lambda_1^a(t)S_1^a - \sigma E_1^a && -\eta^a E_1^a + \eta^{a-1}E_1^{a-1} \\
\dot{A_1^a} &= p^a\sigma E_1^a - \gamma_1 A_1^a && -\eta^a A_1^a + \eta^{a-1}A_1^{a-1} \\
\dot{I_1^a} &= (1-p^a)\sigma E_1^a - \gamma_1 I_1^a && -\eta^a I_1^a + \eta^{a-1}A_1^{a-1} \\
\dot{R_1^a} &= \gamma_1 A_1^a + \gamma_1 I_1^a - \omega R_1^a && -\eta^a R_1^a + \eta^{a-1}R_1^{a-1} \\
\dot{S_2^a} &= \omega R_1^a - \lambda_2^a(t)S_2^a && -\eta^a S_2^a + \eta^{a-1}S_2^{a-1} \\
\dot{E_2^a} &= \lambda_2^a(t)S_2^a - \sigma E_2^a && -\eta^a E_2^a + \eta^{a-1}E_2^{a-1} \\
\dot{A_2^a} &= p^a\sigma E_2^a - \gamma_2 A_2^a && -\eta^a A_2^a + \eta^{a-1}A_2^{a-1} \\
\dot{I_2^a} &= (1-p^a)\sigma E_2^a - \gamma_2 I_2^a && -\eta^a I_2^a + \eta^{a-1}I_2^{a-1} \\
\dot{R_2^a} &= \gamma_2 A_2^a + \gamma_2 I_2^a - \omega R_2^a && -\eta^a R_2^a + \eta^{a-1}R_2^{a-1} \\
\dot{S_3^a} &= \omega R_2^a + \omega R_3^a - \lambda_3^a(t)S_2^a && -\eta^a S_3^a + \eta^{a-1}S_3^{a-1} \\
\dot{E_3^a} &= \lambda_3^a(t)S_2^a - \sigma E_3^a && -\eta^a E_3^a + \eta^{a-1}E_3^{a-1} \\
\dot{A_3^a} &= p^a\sigma E_3^a - \gamma_3 A_3^a && -\eta^a A_3^a + \eta^{a-1}A_3^{a-1} \\
\dot{I_3^a} &= (1-p^a)\sigma E_3^a - \gamma_3 I_3^a && -\eta^a I_3^a + \eta^{a-1}I_3^{a-1} \\
\dot{R_3^a} &= \gamma_3 A_3^a + \gamma_3 I_3^a - \omega R_3^a && -\eta^a R_3^a + \eta^{a-1}R_3^{a-1} \\
\dot{Z^a} &= \sigma(E_0^a + E_1^a + E_2^a + E_3^a)
\end{aligned}
\tag{1}
$$

where an overdot refers to differentiation with respect to t, $\mathbb{1}_1(a)$ is the indicator function (non-zero at $a = 1$). The value of p_R depends on the maternal protection model (see **Section 1.1**) and $\lambda_i^a(t)$ is the force of infection for age group a (see **Section 1.3**). A schematic showing the relationship between each of the epidemiological states variables and the epidemiological parameters is given in **Figure 1**.

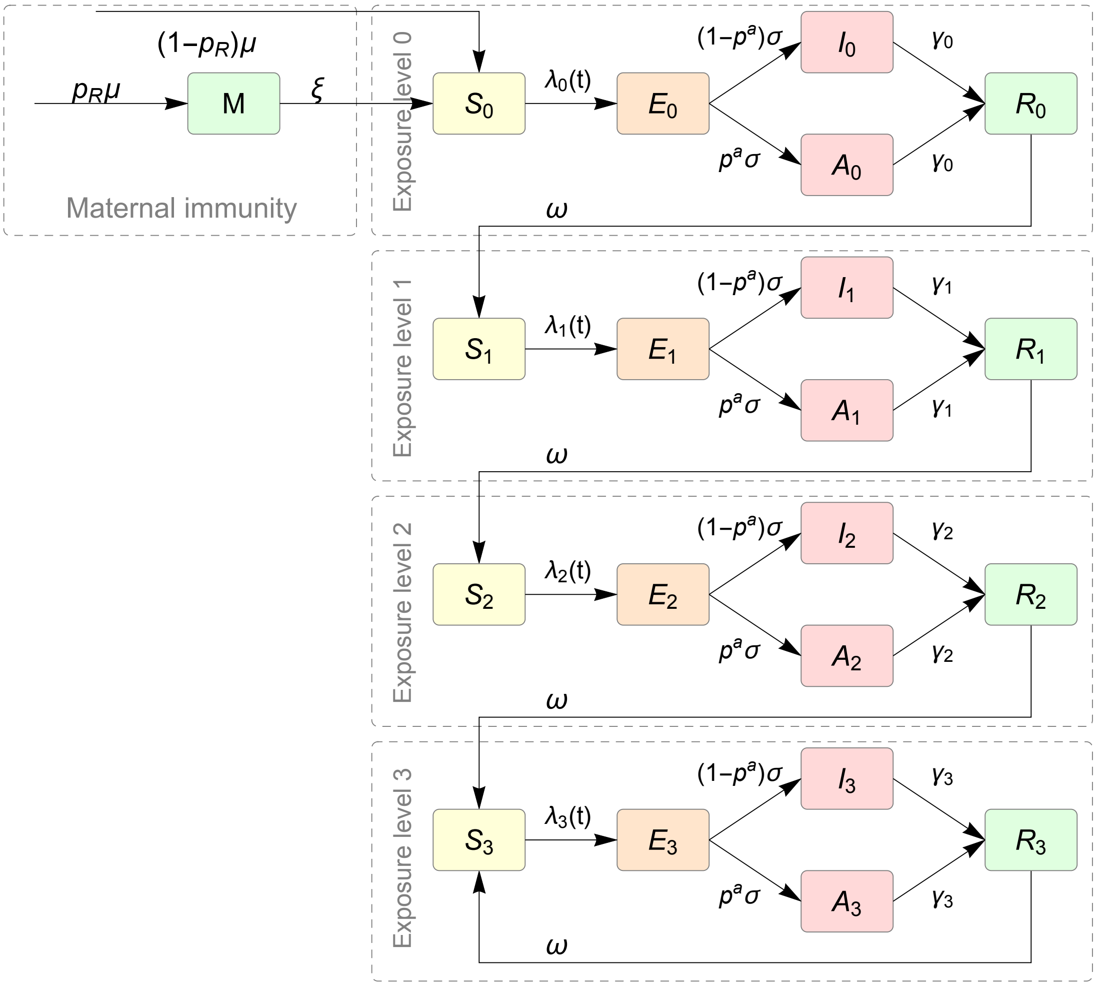

Figure 1: The relationship between the epidemic model state variables (M: protected due to maternal antibodies, S: susceptible, E: exposed but not infectious, I: infectious and symptomatic, A: infectious and asymptomatic, R: recovered and protected) for each of the four exposure levels (subscript $i = 0, 1, 2, 3$). For maternal immunity, the parameters are μ the daily birth rate, p_R the proportion of neonates born with protection and ξ the rate of loss maternal immunity. For each exposure level i, λ_i is the force of infection, σ is the rate of loss exposure to infection, p^a is the probability that an RSV infection is asymptomatic in age group a, γ_i is the rate of loss of infectiousness, and ω is the rate of loss of post-infection immunity.

1.1 Maternal protection model

We considered two different model structures to capture the dynamics of maternal protection. The first, static immunity model, $\mathcal{M}^1$, assumes that all neonates are born with protection, ($p_R = 1$). The second, dynamic immunity model $\mathcal{M}^2$, assumes that the proportion of infants born with protection is equal to the proportion of women of child bearing age (15-44 years) who are in epidemiological state, R at time t, $p_R(t) = \sum_{a=19}^{21} R^a(t) / \sum_{a=19}^{21} N^a$ under the observation that cord titre changes in neonates seasonally, which could influence susceptibility to infection (**Figure 2**).[2]

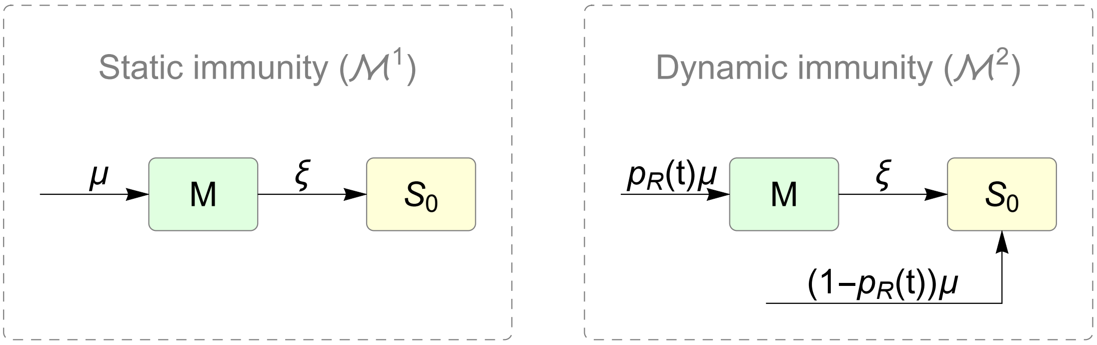

Figure 2: Two models of maternal protection where μ is the daily birth rate, ξ is the rate of loss of maternal-derived immunity, and $p_R(t)$ is the proportion of infants born with protection at time t.

1.2 Contact matrices

To estimate the number of contacts between age group a and b, we combined the results of two contact surveys. The first study (Study A), was conducted as part of the EU funded POLYMOD study—a large pan-European survey with 7,290 participants who recorded 97,904 contacts across all age groups.[3] The second study (Study B), is a smaller study in the United Kingdom with 122 number of participants (all under the age of one year) who recorded 758 contacts.[4] Both studies provided estimates for the number of daily household/non-household contacts and daily physical/conversational contacts made between each age group. Therefore, to estimate the total number of daily physical/conversational contacts made between age group a and b, ($\mathfrak{p}^{a,b}$ and $\mathfrak{c}^{a,b}$ respectively), we used Study A for participants less than 1 years of age, and Study B for older participants. To ensure this symmetry occurs in the contact matrices, we calculated the weighted mean number of contacts made between age a to age group b for conversational contacts (same formula for physical contacts) as:

$$\mathfrak{c}^{a,b} \leftarrow \frac{1}{N^a + N^b}\left(\mathfrak{c}^{a,b}N^a + \mathfrak{c}^{b,a}N^b\right)$$

where N^a is the population size for age group a. The resulting symmetric contact matrices for $\mathfrak{p}^{a,b}$, $\mathfrak{c}^{a,b}$ are plotted in **Figure 3**.

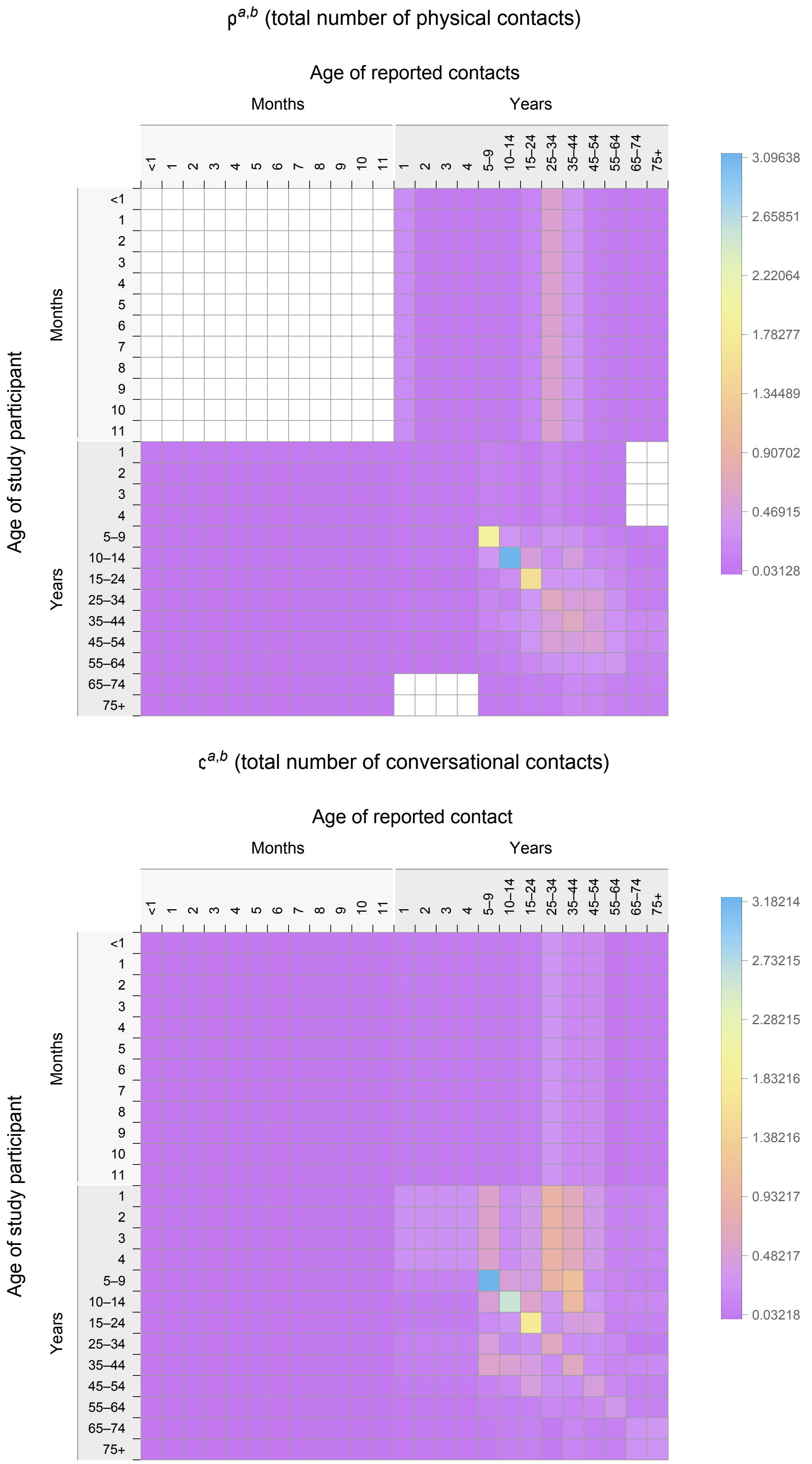

Figure 3: Top: Number of daily physical contacts made between age group a and age group b. Bottom: Number of daily conversational contacts made between age group a and age group b.

1.3 Force of infection

The probability of transmission for a contact made between two age groups is q_p if the contact is physical and $q_p q_c$ if the contact is conversational, where $0 < q_c < 1$ is the reduction in infectiousness of conversation contacts relative to physical contacts. Further, due to climatic factors, we assumed that the probability of transmission is seasonally forced according to a normal distribution, with peak transmission occurring at ϕ, mean b_1 and standard deviation ψ. Finally, because asymptomatic infections are shorter and have a lower viral load then symptomatic infection, we assume the infectiousness of asymptomatic infections is reduced by a factor of $0 < \alpha < 1$. The equation for the force of infection is therefore:

$$\lambda_i^a(t) = q_p(1 + b_1 \exp((t-\phi)^2/(2\psi^2)) \prod_{i'=0}^{i} \delta_{i'} \sum_{b=1}^{25} \frac{(\mathfrak{p}^{a,b} + q_c \mathfrak{c}^{a,b})}{N^b} \left(A_i^b \alpha + I_i^b\right) \tag{2}$$

1.4 Initial conditions

For each age group a, we estimated i) the initial proportion of persons who still have maternally derived immunity, p_ξ^a, (by assuming loss of immunity is exponentially distributed with rate ξ) and ii) the initial proportion of persons who have experienced k number of previous infections p_k^a (assuming acquisition of infection is Poisson distributed with rate 1 year). The initial proportion of persons in each exposure level who are not infected is therefore given by $(1 - l_1)$, and of this proportion, l_2 are in epidemiological group R_i, with the rest in epidemiological group S_i. Of the infected proportion, l_1, the initial proportion in state E, is the average amount of time within that epidemic group $(\sigma/(\sigma + \gamma_i))$. Following a similar argument, the formulae for the initial proportion of the infected persons who are in the asymptomatic and symptomatic state is $\sigma/(\sigma + \gamma_i)p^a$ and $\sigma/(\sigma + \gamma_i)(1 - p^a)$ respectively (**Figure 4**).

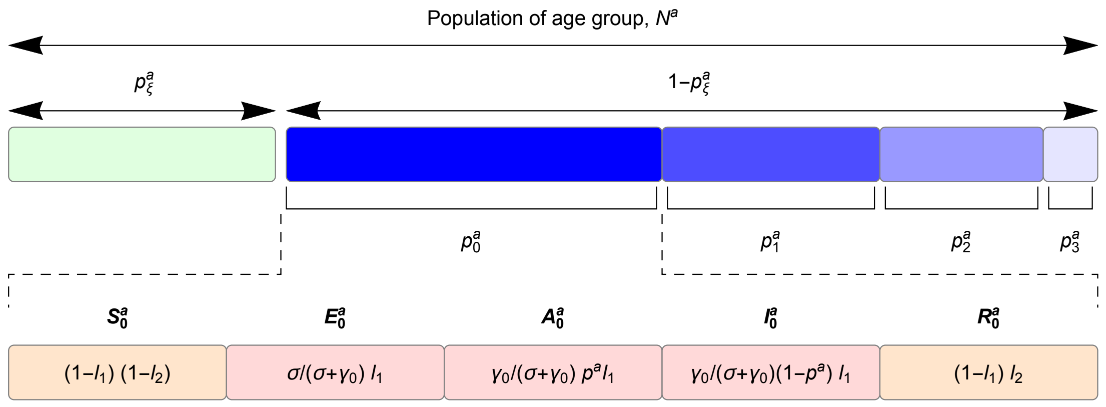

Figure 4: The formulae for calculating the initial conditions of the epidemic state variables using the parameters, p_ξ^a, the initial proportion of persons in a demographic group with maternal protection, and p_i^a the initial proportion of persons in a demographic group in exposure group i. (see **Equations 4–5** for formulae).

The equations for the initial conditions are:

$$
\begin{array}{ll}
M^a(0) = Np^a_\xi & \\
S^a_0(0) = \left[N(1-p^a_\xi)p^a_0\right](1-l_1)(1-l_2) & E^a_0(0) = \left[N(1-p^a_\xi)p^a_0\right]\left(\frac{\sigma}{\gamma_0+\sigma}\right)l_1 \\
A^a_0(0) = \left[N(1-p^a_\xi)p^a_0\right]\left(\frac{\gamma_0}{\gamma_0+\sigma}\right)(p^a)l_1 & I^a_0(0) = \left[N(1-p^a_\xi)p^a_0\right]\left(\frac{\gamma_0}{\gamma_0+\sigma}\right)(1-p^a)l_1 \\
R^a_0(0) = \left[N(1-p^a_\xi)p^a_0\right](1-l_1)l_2 & \\
S^a_1(0) = \left[N(1-p^a_\xi)p^a_1\right](1-\delta_1 l_1)(1-l_2) & E^a_1(0) = \left[N(1-p^a_\xi)p^a_1\right]\left(\frac{\sigma}{\gamma_1+\sigma}\right)\delta_1 l_1 \\
A^a_1(0) = \left[N(1-p^a_\xi)p^a_1\right]\left(\frac{\gamma_1}{\gamma_1+\sigma}\right)(p^a)\delta_1 l_1 & I^a_1(0) = \left[N(1-p^a_\xi)p^a_1\right]\left(\frac{\gamma_1}{\gamma_1+\sigma}\right)(1-p^a)\delta_1 l_1 \\
R^a_1(0) = \left[N(1-p^a_\xi)p^a_1\right](1-\delta_1 l_1)l_2 & \\
S^a_2(0) = \left[N(1-p^a_\xi)p^a_2\right](1-\delta_2 l_1)(1-l_2) & E^a_2(0) = \left[N(1-p^a_\xi)p^a_2\right]\left(\frac{\sigma}{\gamma_2+\sigma}\right)\delta_2 l_1 \\
A^a_2(0) = \left[N(1-p^a_\xi)p^a_2\right]\left(\frac{\gamma_2}{\gamma_2+\sigma}\right)(p^a)\delta_2 l_1 & I^a_2(0) = \left[N(1-p^a_\xi)p^a_2\right]\left(\frac{\gamma_2}{\gamma_2+\sigma}\right)(1-p^a)\delta_2 l_1 \\
R^a_2(0) = \left[N(1-p^a_\xi)p^a_2\right](1-\delta_2 l_1)l_2 & \\
S^a_3(0) = \left[N(1-p^a_\xi)p^a_3\right](1-\delta_3 l_1)(1-l_2) & E^a_3(0) = \left[N(1-p^a_\xi)p^a_3\right]\left(\frac{\sigma}{\gamma_3+\sigma}\right)\delta_3 l_1 \\
A^a_3(0) = \left[N1-p^a_\xi)p^a_3\right]\left(\frac{\gamma_3}{\gamma_3+\sigma}\right)(p^a)\delta_3 l_1 & I^a_3(0) = \left[N(1-p^a_\xi)p^a_3\right]\left(\frac{\gamma_3}{\gamma_3+\sigma}\right)(1-p^a)\delta_3 l_1 \\
R^a_3(0) = \left[N(1-p^a_\xi)p^a_3\right](1-\delta_3 l_1)l_2 & Z^a(0) = 0
\end{array}
\tag{3}
$$

where $N = N^a$; the equation for p^a_ξ, the initial proportion of persons in age group, a (age range, $[n^{a-1}, n^a]$, $n^{a-1} < n^a$) who still have maternal protection given a rate of loss of maternal protection parameter, ξ, is:

$$
p^a_\xi = \frac{1}{(n^a - n^{a-1})} \int_{n^{a-1}}^{n^a} \exp(-365\xi x)\, dx \tag{4}
$$

and the equations for p^a_k, the initial proportion of persons in age group a who have experienced k number of previous infections assuming no cumulative protection follows a Poisson distribution, is:

$$
\begin{array}{l}
p^a_k = \frac{1}{(n^a - n^{a-1})} \int_{n^{a-1}}^{n^a} \frac{(x)^k \exp(-x)}{k!} dx, \quad k = 0,1,2 \\
p^a_3 = 1 - (p^j_0 + p^j_1 + p^j_2)
\end{array}
\tag{5}
$$

1.5 Model output

The output of the epidemic model is the number of new infections $Z^{\mathcal{M}^m,a}_{w_t}$ in age group a, maternal model m, per week w_t and the formula is:

$$
Z^{\mathcal{M}^m,a}_{w_t} = \frac{Z^{\mathcal{M}^m,a}(t)}{dt}\Bigg|_{t=w_{k-1}}^{t=w_k} \tag{6}
$$

where $Z^{\mathcal{M}^m,a}(t)$ is the cumulative number of new infections at time t under maternal immunity model m.

2 Parameterisation of prior distributions

2.1 Duration of immunity

It is unclear what the period of naturally-acquired immunity is for RSV, however, observational cohort studies suggest that reinfection is possible after 60 days and it is also reasonable to assume that some hosts are susceptible again at the start of an RSV season (on average 200 days later).[5,6] Therefore, we assumed the prior distribution for the duration of protection of $\mathcal{N}(130, 35)$ so that the 95% CI corresponds with 60 and 200 days. For duration of maternal protection, having a higher baseline cord blood antibody level for RSV at birth provides i) a significant decrease in disease incidence during in the first 6 months of life[7–9] and ii) a decrease in risk of hospital admission.[2] Therefore, we assumed the duration of maternal protection can be no shorter than 14 days and no longer than 6 months, giving a prior of $\mathcal{U}(14, 180)$.

2.2 Duration of symptomatic infection

For the prior for the duration of the latency period ($1/\sigma$), we used an experimental challenge study[10] to estimate the mean and standard deviation as 4.0 and 1.5 days respectively. Using the formula

$$Gamma\left(\frac{\mu^2}{s^2}, \frac{s^2}{\mu}\right) \tag{7}$$

where μ is the mean and s^2 is the variance, the fitted distribution for $1/\sigma$ is Gamma$(7.111, 0.563)$. To ensure that the duration of infection decreased with repeated exposure, we found prior distributions for the duration of primary infection, $1/\gamma_0$, and the decrease in duration of infection relative to the previous infection, g_i such that $\gamma_1 \equiv \gamma_0(g_1)^{-1}$, $\gamma_2 \equiv \gamma_0(g_1g_2)^{-1}$, and $\gamma_3 \equiv \gamma_0(g_1g_2g_3)^{-1}$. The mean and 95% confidence interval for primary and subsequent infection from a prospective cohort study were the convolution distributions:[11] 5.1 (95% CI 4.2–6.2) + $\mathcal{U}(0, 7)$ and 4.0 (95% CI 3.3–4.9) + $\mathcal{U}(0, 7)$ respectively where the uniform distribution arises to account for left-censoring in weekly collection protocol. The empirical sample for the prior distributions for $1/\gamma_0$ is found by sampling from 5.1 (95% CI 4.2–6.2) + $\mathcal{U}(0, 7)$ and fitting the sample to a probability distribution. The method of fitting an empirical sample to a probability distribution, we refer to as **Fitting procedure 1**:

Fitting procedure 1 To fit an empirical distribution to a probability distribution we use the maximum likelihood method to estimate the parameters of the i) Gamma(k, θ), ii) $\mathcal{LN}(\mu, \sigma)$, and iii) $W(\lambda, k)$, and choose the probability distribution with the highest likelihood.

Fitting procedure 1 gives a probability distribution of $W(4.137, 8.303)$ for $1/\gamma_0$. For g_1 we divided the samples from 5.1 (95% CI 4.2–6.2) + $\mathcal{U}(0, 7)$ by the samples from 4.0 (95% CI 3.3–4.9) + $\mathcal{U}(0, 7)$ and used **Fitting Procedure 1** on the resulting sample to get a probability distribution of $W(34.224, 0.879)$ for g_1. For g_2, we used an experimental reinfection study[10] to find a mean and standard deviation for γ_2 of 3.6 and 1.1 days respectively (Gamma$(10.71, 0.34)$ from **Equation 7**). Dividing ordered samples from this distribution by the ordered empirical sample for γ_0 multiplied by $(g_1)^{-1}$ gives an empirical sample for the prior distribution for g_2 which, from **Fitting procedure 1**, has a probability distribution $\mathcal{LN}(-0.561, 0.163)$. As there is no evidence to suggest the duration of infection decreases further after tertiary infection, $g_3 = 1$.

2.3 Susceptibility to infection

The prior distribution for the reduction in susceptibility to infection, δ_i, assuming i number of previous infections, is determined using two prospective cohort studies[12,13] which estimated the average proportion of individuals who become infected when challenged with RSV for secondary, tertiary and subequent infections, relative to their previous infection, as 0.757, 0.878 and 0.322 respectively (with sample sizes of 47, 26 and 19). Using the formula

$$\mathcal{B}(\mu n, (1-\mu)n) \tag{8}$$

where μ is the mean, and n is the sample size, we estimated the probability distributions for these observations as $\mathcal{B}(35.583, 11.417)$, $\mathcal{B}(22.829, 3.171)$ and $\mathcal{B}(6.117, 12.882)$ for susceptibility to secondary and tertiary and subsequent infection, relative to previous infection.

2.4 Asymptomatic infection

The proportion of infections which are asymptomatic is estimated from a prospective cohort study[14] which showed, for ages <1, 1-4, 5-14, and 15 years and over, the mean probability of asymptomatic infection is 0.091, 0.173, 0.521, and 0.765, for the sample sizes is 33, 52, 73, and 47 respectively (giving $\mathcal{B}(3.003, 29.997)$, $\mathcal{B}(8.996, 43.004)$, $\mathcal{B}(38.033, 34.967)$ and $\mathcal{B}(35.955, 11.045)$ from the formula **Equation 8**). Though exiting studies have estimated the difference in viral load and the duration of shedding between asymptomatic and symptomatic infection, it is unclear how these differences alter the infectiousness of a host.[14] Therefore, as there is no strong evidence otherwise, we assumed the prior distributions for α of $\mathcal{U}(0, 1)$.

2.5 Transmission and initial parameters

Finally, as they cannot be estimated from epidemiological data, the prior distributions for the transmission probability per contact physical contact q_p, relative reduction in transmission due to conversational contact q_c, the relative seasonal amplitude b_1, the offset ϕ and the width of heightened transmissive season ψ all have prior distributions of $\mathcal{U}(0, 1)$. A summary of all the prior distributions described above associated with the epidemic model is given in **Table 3**.

	Parameter	Value	Source
Duration of immunity			
$1/\xi$	Maternally-derived (days)	$\mathcal{U}(14, 180)$	[7–9]
$1/\omega$	Post-infection (days)	$\mathcal{N}(135, 35)$	[5,6]
Duration of symptomatic infection			
$1/\sigma$	Exposure (days)	$\text{Gamma}(7.111, 0.563)$	[10]
$1/\gamma_0$	Primary infection (days)	$W(4.137, 8.303)$	[11]
g_1	Proportional decrease between secondary and primary infection	$W(34.224, 0.879)$	[11]
g_2	Proportional decrease between tertiary and secondary infection	$\mathcal{LN}(-0.561, 0.163)$	
Susceptibility			
δ_1	Relative susceptibility to secondary infection, relative to primary infection	$\mathcal{B}(35.583, 11.417)$	[12]
δ_2	Relative susceptibility to tertiary infection, relative to secondary infection	$\mathcal{B}(22.829, 3.171)$	[12]
δ_3	Relative susceptibility to subsequent infections after third infection, relative to tertiary infection	$\mathcal{B}(6.117, 12.882)$	[12]
Asymptomatic infection			
$p^{<1}$	Proportion asymptomatic (<1 years)	$\mathcal{B}(3.003, 29.997)$	[14]
p^{1-4}	Proportion asymptomatic (1–4 years)	$\mathcal{B}(8.996, 43.004)$	[14]
p^{5-14}	Proportion asymptomatic (5–14 years)	$\mathcal{B}(38.033, 34.967)$	[14]
$p^{>15}$	Proportion asymptomatic (15+ years)	$\mathcal{B}(35.955, 11.045)$	[14]
α	Reduction in infectiousness	$\mathcal{U}(0, 1)$	—
Transmission parameters			
q_p	Probability of transmission of RSV per physical contact.	$\mathcal{U}(0, 1)$	—
q_s	Reduction in transmission due to conversational contact	$\mathcal{U}(0, 1)$	—
b_1	Relative amplitude	$\mathcal{U}(0, 1)$	—
ϕ	Seasonal offset	$\mathcal{U}(0, 1)$	—
ψ	Width of seasonal peak	$\mathcal{U}(0, 1)$	—
Initial parameters (at $t = 0$, age group a)			
l_1	Initial proportion infected	$\mathcal{U}(0, 1)$	—
l_2	Initial proportion of non-infected individuals who are protected	$\mathcal{U}(0, 1)$	—

Table 3: Prior distributions of the parameters in the transmission model. Subscript i indicates exposure level and superscript a indicates age group.

3 Model-fitting

3.1 Detection model

The virological surveillance data used to calibrate the transmission model is the Respiratory DataMart System (RDMS). RDMS is a laboratory-based virological sentinel surveillance system, which systematically collects data on the number of RSV positive and negative clinical respiratory samples from 14 Public Health England (PHE) and National Health Service (NHS) laboratories in England.[15] From RDMS, we extracted the total number of weekly laboratory-confirmed cases of RSV from July 2010 and up until June 2017 for each age group. The number of positive samples for age group a and week number w_t is given by $d^a_{w_t} \in \mathcal{D}$, where $\mathcal{D}$ is the set of all samples.

Only a small proportion of the total RSV infections will be detected by the RDMS. This is because RSV infections which are included are only those in which the infected individual:

1. acquired infection in a region which is covered by the surveillance system
2. consulted healthcare at some clinical interface
3. the health care profession offering a test
4. the test is accurate in detecting the RSV virus

As severity of RSV infection depends on age, points 2) and 3) imply that the proportion of total RSV infections which are present in the RDMS is likely to be dependent on age. Therefore, we assumed that the per-infection detection probability by the RDMS surveillance system, ϵ^a, could be dependent on age (note that $Z^a_{w_t}\epsilon^a \approx d^a_{w_t}$).

Due to lack of direct information for estimates of the detection probability, we first estimated an approximate value, $\bar{\epsilon}^a$ by dividing the proportion of the population which are reported in the dataset p^a_+ by an estimate for the attack rate in age group, r^a (**Figure 5**). The estimate for the attack rate is found from a prospective cohort study[16] for children less than 5 years, and for individuals greater than 5 years we used the attack rate from the aforementioned prospective cohort study for the first year, under the assumption all infants are fully susceptibility, and multiplied it by the prior distribution for the relative reduction in susceptibility δ_i. The approximate values for the detection probability, $\bar{\epsilon}^a = p^a_+/r^a$, are plotted in **Figure 5**. By defining the total number of positive samples for age group a per year from RDMS as D^a, the weighted proportion of samples for each age group is given by $w^a = D^a/\Sigma_a D_a$.

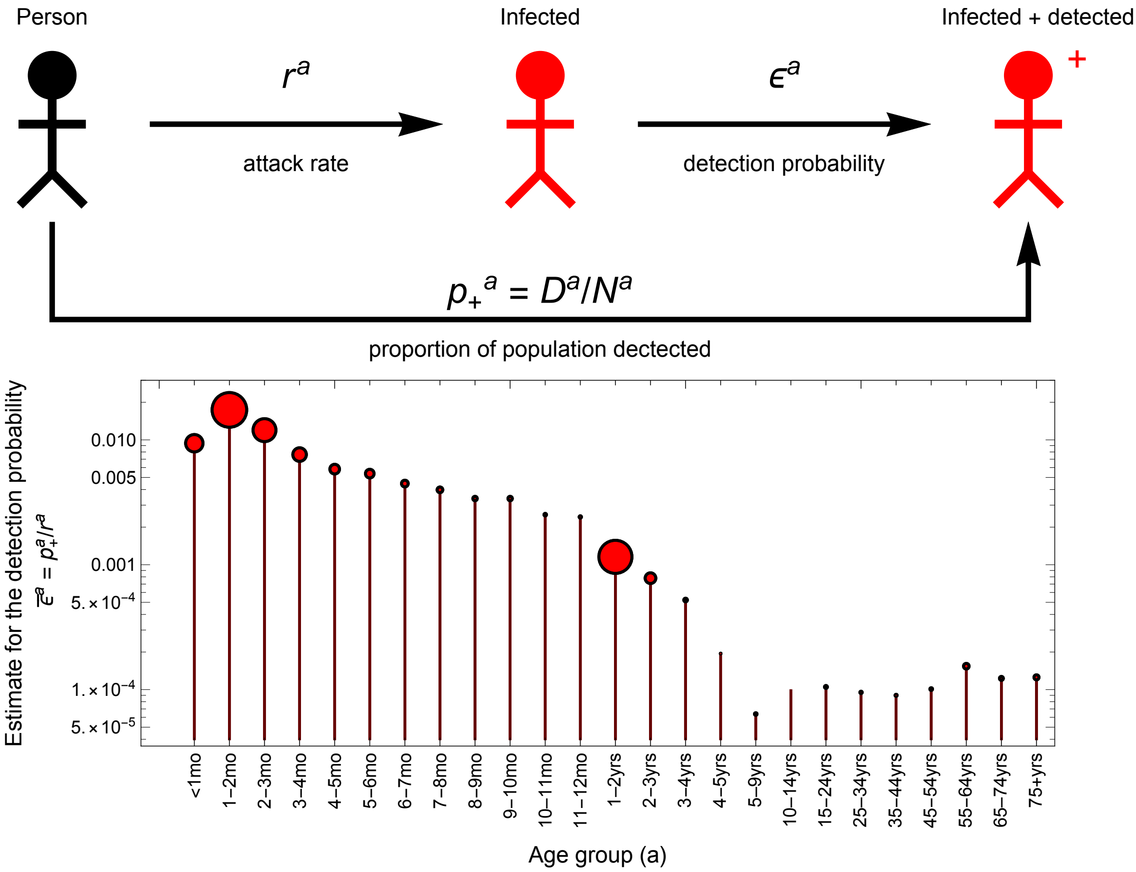

Figure 5: Top: Schematic showing the multiplicative relationship in age group a between the estimated attack rate r^a, the detection probability ϵ^a, and the proportion of the population caught in the RDMS surveillance dataset, p_+^a. Bottom: For each age group a, this plot shows the estimated value for the detection probability $\bar{\epsilon}^a$, and the number of positive RSV samples from the RDMS dataset D^a which is proportional with the radius of the point marker.

Assuming that each age group has a unique detection probability could over fit the model, however, using too few detection probabilities lead to a poorly fitted model. We chose the optimal number of age dependent detection rates by performing a formal model comparison using Akaike Information Criteria (AIC) to choose between 5 models which vary in the number of detection probabilities used between ages 0-4 years. The first age structure ($\mathcal{E}^1$) assumed the same detection probability value for all 0–4 year olds. The second, third and fourth structures ($\mathcal{E}^2$, $\mathcal{E}^3$ and $\mathcal{E}^4$) assumed that the 0–4 age groups is parameterised by 2, 3 and 4 different detection probabilities. To find the optimal age stratification for each of these three structures, we fitted the values of $\bar{\epsilon}^j$ to a discrete-valued function using a weighted least squares method (using the weights w^j) for all possible stratifications of this age group and then chose the age stratification with the smallest corresponding AIC. This method gave the optimal age stratifications of {0–2mo,3mo–4yrs}, {0–2mo, 3–7mo, 8mo–4yrs} and {0–2mo, 3–5mo, 6–11mo,1–4yrs} for the three structures respectively. For the fifth structure, we assumed that the values of detection probability are parameterised according to an exponential decay $\exp(ax + b)$, where a and b are parameters be estimated.

For detection models $\mathcal{E}^j, j = \{1, 2, 3, 4\}$, the prior distribution for each of the detection probabilities ϵ^j, were found by calculating the weighted mean and standard deviation of the estimated detection probabilities values $\bar{\epsilon}^j$ contained within the age range of the stratification and then fitting these moments (through **Equation 7**) to a Gamma distribution. For $\mathcal{E}^5$, the detection probability for age group j, is given by fitting a non-linear weighted least squares with the exponential func-

tion of the form $\exp(ax+b)$ to the estimated detection probabilities values between 0 and 4 years. The mean and standard deviations of the parameters of the fitted exponential (a and b) are then then assumed to follow a normal distribution. A summary of all the age stratifications and prior distributions for all five of the model structures are given in **Table 4**.

Parameter		Prior distribution	Source
Detection model structure 1, $\mathcal{E}^1 = \{\epsilon^1_{S_1}, \epsilon^2_{S_1}, \epsilon^3_{S_1}\}$			
$\epsilon^1_{S_1}$	0–4yrs	Gamma(1.4278, 0.0050)	$\overline{\epsilon}^a$
Detection model structure 2, $\mathcal{E}^2 = \{\epsilon^1_{S_2}, \epsilon^2_{S_2}, \epsilon^3_{S_2}, \epsilon^4_{S_2}\}$			
$\epsilon^1_{S_2}$	0–2mo	Gamma(10.9978, 0.0013)	$\overline{\epsilon}^a$
$\epsilon^2_{S_2}$	3mo–4yrs	Gamma(1.7757, 0.0018)	$\overline{\epsilon}^a$
Detection model structure 3, $\mathcal{E}^3 = \{\epsilon^1_{S_3}, \epsilon^2_{S_3}, \epsilon^3_{S_3}, \epsilon^4_{S_3}, \epsilon^5_{S_3}\}$			
$\epsilon^1_{S_3}$	0–2mo	Gamma(10.9978, 0.0013)	$\overline{\epsilon}^a$
$\epsilon^2_{S_3}$	3–8mo	Gamma(11.9721, 0.00045)	$\overline{\epsilon}^a$
$\epsilon^3_{S_3}$	9mo–4yrs	Gamma(2.16447, 0.00063)	$\overline{\epsilon}^a$
Detection model structure 4, $\mathcal{E}^4 = \{\epsilon^1_{S_4}, \epsilon^2_{S_4}, \epsilon^3_{S_4}, \epsilon^4_{S_4}, \epsilon^5_{S_4}, \epsilon^6_{S_4}\}$			
$\epsilon^1_{S_4}$	0–2mo	Gamma(10.9978, 0.0013)	$\overline{\epsilon}^a$
$\epsilon^1_{S_4}$	3–6mo	Gamma(27.1392, 0.00024)	$\overline{\epsilon}^a$
$\epsilon^3_{S_4}$	7–11mo	Gamma(19.8873, 0.00018)	$\overline{\epsilon}^a$
$\epsilon^4_{S_4}$	1–4yrs	Gamma(7.64267, 0.00012)	$\overline{\epsilon}^a$
Detection model structure 5, $\mathcal{E}^5 = \{\epsilon^1_{S_5}, \epsilon^2_{S_5}, \dots \epsilon^{17}_{S_5}, \epsilon^{18}_{S_5}\}, \epsilon^j_{S_5} = \exp(a + b * j)$			
a	0–4yrs	$\mathcal{N}(-3.9885, 0.1357)$	$\overline{\epsilon}^a$
b		$\mathcal{N}(-0.1794, 0.0413)$	$\overline{\epsilon}^a$
Common to all model structures, $\lvert\mathcal{E}^k\rvert = A_k$			
$\epsilon^{A_{k-1}}_{S_k}$	5–54yrs	Gamma(35.0678, 2.61628×10^{-6})	$\overline{\epsilon}^a$
$\epsilon^{A_k}_{S_k}$	55+ yrs	Gamma(59.2461, 2.28079×10^{-6})	$\overline{\epsilon}^a$

Table 4: Prior distributions for the parameters in the five detection models.

3.2 Calibration

We performed inference on the parameter set:

$$\theta^{m,e} = \mathcal{M}^m \cup \mathcal{E}^e$$

where $m \in \{1,2\}$ and $e \in \{1,2,3,4,5\}$ are the possible maternal protection and detection model structures. For each model structure, the transmission model estimated the number of new infections per week $Z^a_{w_t}$ and the detection model estimated the age-dependent probability of being reported in the RDMS dataset, ϵ^a. We assume that year-to-year changes in the number of RSV positive samples are due to i) changes in sampling protocol, ii) hospital admission thresholds being lowered (particularly in the younger infants) and/or iii) failure to manage these acute illnesses in the community care setting.[17] Therefore, to account for these year-to-year changes we normalise the number of RSV positive samples in age group a during year y relative to year 7 (2016–17) so that each year has the same total number of positive samples in age group a. Mathematically, for the number of positive samples $d^a_{w_t}$ for age group a during week number w_t, we define

$$D^a_y = \sum_{t=1+52\times6(y-1)}^{52+52\times6(y-1)} d^a_{w_t} \tag{9}$$

Then, the normalised data $\bar{d}^a_{w_t}$ during year y, is given by

$$\bar{d}^a_{w_t} = \frac{d^a_{w_t} D^a_7}{D^a_y} \tag{10}$$

By treating each infection in age group a as a Bernoulli trial, which has probability of success (being detected in the normalised RDMS dataset $\mathcal{D}$) of ϵ^a, the likelihood function for the parameter set $(\theta^{m,e})$ for week, w_t and age group a is given by the binomial distribution $\bar{d}^a_{w_t} \sim \text{Bin}(Z^{\mathcal{M}^m,a}_{t_w}, \epsilon^a)$. Fitting the output for each age group over to seven years of weekly incidence data, the full likelihood is the product of each age and weekly binomial likelihood function:

$$\mathcal{L}(\mathcal{D}|\theta^{m,c}) = \mathcal{L}(\mathcal{D}|\mathcal{M}^m, \mathcal{E}^c) = \prod_{a=1}^{25} \prod_{t=1}^{7*52} \text{Bin}(Z^{\mathcal{M}^m a}_{w_t}, \epsilon^a)$$

Using this likelihood and the prior distributions, the posterior distributions for the parameters in the model are determined using an adaptive parallel tempering Metropolis Hastings algorithm with a temperature ladder consisting of 12 chains and an adaptive covariance matrix.[18] The proposal distribution was a multivariate truncated normal distribution ($\mathcal{TN}$), with the boundaries of the distributions equal to the support for each parameter. Thus, for each of the 12 chains, given a Markov chain of length, i, $\{\theta_t\}^i_{t=0}$ the equation of the acceptance probability of a new position, $\theta' \sim \mathcal{TN}(\theta_i, \Sigma_i)$ is

$$a(\theta_i, \theta') = \frac{\mathcal{L}(\mathcal{D}|\theta')p(\theta')}{\mathcal{L}(\mathcal{D}|\theta_i)p(\theta_i)} \frac{\mathcal{TN}(\theta_i|\theta', \Sigma_i)}{\mathcal{TN}(\theta'|\theta_i, \Sigma_i)} \tag{11}$$

3.3 Model choice

To determine which of the model structures (maternal protection model m and detection model e) best estimates the incidence of RSV given the RDMS data, we calculated a Deviance Information Criterion (DIC) given by

$$\text{DIC}^{m,c} = -2(2\overline{\mathcal{L}(\mathcal{D}|\theta^{m,c})} - \mathcal{L}(\mathcal{D}|\bar{\theta}^{m,c})) \tag{12}$$

where $\mathcal{L}(\mathcal{D}|\bar{\theta}))$ is the likelihood of the mean of the posterior samples and $\overline{\mathcal{L}(\mathcal{D}|\theta)}$ is the mean of the likelihood of the posteriors samples.

3.4 Posterior distributions

Each of the 12 Markov chains ran for 50,000 steps, where the first 25,000 steps were the burn-in and the final 25,000 steps were the empirical samples for the posterior distributions for each of the parameters. The final posterior samples were thinned every 20 steps, given a empirical sample of 1,250 values for the joint posterior distribution.

3.5 Implementation

The transmission model ODEs were solved using the Euler method in Ascent package in C++, using a time step of 1 day over a 8 year period, (1 year to reach a stead state and 7 years to calculate the likelihood).

The binomial likelihood function leads to computationally unmanageable values, therefore we consider the log likelihood. The equation for the log likelihood function is therefore:

$$\log \mathcal{L}(\mathcal{D}|\theta) \approx \begin{cases} \sum_{a=1}^{25} \sum_{t=1}^{52\times7} -Z^a_{w_t}\epsilon^a, & \text{when } \bar{d}^a_t = 0 \\ \sum_{a=1}^{25} \sum_{t=1}^{52\times7} \bar{d}^a_t \log(Z^a_{w_t}\epsilon^j) - nZ^{a,\theta}_{w_t}\epsilon^j - \sum_{k=1}^{\bar{d}^a_t} \log(k), & \text{when } \bar{d}^a_t > 0 \end{cases} \tag{13}$$

and the acceptance probability can be calculated:

$$a(\theta_i, \theta') = \exp(\log \mathcal{L}(\mathcal{D}|\theta') + \log(p(\theta')) - \log \mathcal{L}(\mathcal{D}|\theta_i) - \log(p(\theta_i)) + \overbrace{\log(\mathcal{TN}(\theta_i|\theta', \Sigma_i) - \mathcal{TN}(\theta'|\theta_i, \Sigma_i))}^{\text{Correction constant}}) \tag{14}$$

Evaluating the correction constant is computationally difficult as it involves evaluating two points from multivariate truncated normal distributions in a high number of dimensions. Therefore, to evaluate a this term, we used an expected propagation method outlined in Cunningham et al.[19]

4 Intervention model

4.1 Palivizumab programme

In order to evaluate to the impact of the Palivizumab programme we stratified the infants according to whether they are Palivizumab eligible (VHR) or not (indicated by the superscript r). To estimate the proportion of infants who are eligible for Palivizumab in age group a ($p^{a,VHR}$), we first estimated the number of infants who receive Palivizumab per season in England from the number of Palivizumab units sold.[20] Then, we determined the age distribution using estimates for of infants who are prematurely born with Chronic Lung Disease (CLD) and Chronic Heart Disease (CHD) by gestational age in the UK[21,22] and the eligibility criteria for Palivizumab in UK.[23] This gives an estimate for the proportion of Palivizumab eligible persons of 0.00348, 0.00227 for infants aged < 1 and 1 month of age, 0.00066 for infants aged 2-5 months, 0.00002 for infants aged 5-8 months and zero otherwise. The Palivizumab programme is given to all very-high-risk neonates between October and February and assumes a 90% coverage (2,128 courses).

The ODEs of the Palivizumab programme for age group a and clinical-risk group r are:

$$
\begin{array}{rcll}
 & & \overbrace{\hphantom{p_R\mu p^{a,r}\mathbb{1}_1(a)(1-\phi^{a,r}_{P,pal})-\xi M^{a,r}}}^{\text{Transmission terms}} & \overbrace{\hphantom{-\eta^a M^{a,r}+\eta^{a-1}M^{a-1}p^{a,r}(1-\phi^{a,r}_{P,pal})}}^{\text{Ageing terms}} \\
\dot{M^{a,r}} & = & p_R\mu p^{a,r}\mathbb{1}_1(a)(1-\phi^{a,r}_{P,pal})-\xi M^{a,r} & -\eta^a M^{a,r}+\eta^{a-1}M^{a-1}p^{a,r}(1-\phi^{a,r}_{P,pal}) \quad \overbrace{\hphantom{+\dot{V_P^{a,r}}\omega_{pal}}}^{\textbf{Palivizumab terms}} \\
\dot{S_0^{a,r}} & = & (1-p_R)\mu p^{a,r}\mathbb{1}_1(a)(1-\phi^{a,r}_{P,pal})+\xi M^{a,s}-\lambda_0^{a,r}(t)S_0^{a,r} & -\eta^a S_0^{a,r}+\eta^{a-1}S_0^{a-1}p^{a,r}(1-\phi^{a,r}_{P,pal}) \quad +\dot{V_P^{a,r}}\omega_{pal} \\
\dot{E_0^{a,r}} & = & \lambda_0^{a,r}(t)S_0^{a,s}-\sigma E_0^{a,r} & -\eta^a E_0^{a,r}+\eta^{a-1}E_0^{a-1}p^{a,r}(1-\phi^{a,r}_{P,pal}) \\
\dot{A_0^{a,r}} & = & p^a\sigma E_0^{a,r}-\gamma_0 A_0^{a,r}\rho & -\eta^a A_0^{a,r}+\eta^{a-1}A_0^{a-1}p^{a,r}(1-\phi^{a,r}_{P,pal} \\
\dot{I_0^{a,r}} & = & (1-p^a)\sigma E_0^{a,r}-\gamma_0 I_0^{a,r} & -\eta^a I_0^{a,r}+\eta^{a-1}I_0^{a-1}p^{a,r}(1-\phi^{a,r}_{P,pal}) \\
\dot{R_0^{a,r}} & = & \rho\gamma_0 A_0^{a,r}+\gamma_0 I_0^{a,r}-\omega R_0^{a,r} & -\eta^a R_0^{a,r}+\eta^{a-1}R_0^{a-1}p^{a,r}(1-\phi^{a,r}_{P,pal}) \\
\dot{S_1^{a,r}} & = & \omega R_0^{a,r}-\lambda_1^{a,r}(t)S_1^{a,r} & -\eta^a S_1^{a,r}+\eta^{a-1}S_1^{a-1}p^{a,r}(1-\phi^{a,r}_{P,pal}) \\
\dot{E_1^{a,r}} & = & \lambda_1^{a,r}(t)S_1^{a,r}-\sigma E_1^{a,r} & -\eta^a E_1^{a,r}+\eta^{a-1}E_1^{a-1}p^{a,r}(1-\phi^{a,r}_{P,pal}) \\
\dot{A_1^{a,r}} & = & p^a\sigma E_1^{a,r}-\gamma_1 A_1^{a,r}\rho & -\eta^a A_1^{a,r}+\eta^{a-1}A_1^{a-1}p^{a,r}(1-\phi^{a,r}_{P,pal}) \\
\dot{I_1^{a,r}} & = & (1-p^a)\sigma E_1^{a,r}-\gamma_1 I_1^{a,r} & -\eta^a I_1^{a,r}+\eta^{a-1}A_1^{a-1}p^{a,r}(1-\phi^{a,r}_{P,pal}) \\
\dot{R_1^{a,r}} & = & \rho\gamma_1 A_1^{a,r}+\gamma_1 I_1^{a,r}-\omega R_1^{a,r} & -\eta^a R_1^{a,r}+\eta^{a-1}R_1^{a-1}p^{a,r}(1-\phi^{a,r}_{P,pal}) \\
\dot{S_2^{a,r}} & = & \omega R_1^{a,r}-\lambda_2^{a,r}(t)S_2^{a,r} & -\eta^a S_2^{a,r}+\eta^{a-1}S_2^{a-1}p^{a,r}(1-\phi^{a,r}_{P,pal}) \\
\dot{E_2^{a,r}} & = & \lambda_2^{a,r}(t)S_2^{a,r}-\sigma E_2^{a,r} & -\eta^a E_2^{a,r}+\eta^{a-1}E_2^{a-1}p^{a,r}(1-\phi^{a,r}_{P,pal}) \\
\dot{A_2^{a,r}} & = & p^a\sigma E_2^{a,r}-\gamma_2 A_2^{a,r}\rho & -\eta^a A_2^{a,r}+\eta^{a-1}A_2^{a-1}p^{a,r}(1-\phi^{a,r}_{P,pal}) \\
\dot{I_2^{a,r}} & = & (1-p^a)\sigma E_2^{a,r}-\gamma_2 I_2^{a,r} & -\eta^a I_2^{a,r}+\eta^{a-1}I_2^{a-1}p^{a,r}(1-\phi^{a,r}_{P,pal}) \\
\dot{R_2^{a,r}} & = & \rho\gamma_2 A_2^{a,r}+\gamma_2 I_2^{a,r}-\omega R_2^{a,r} & -\eta^a R_2^{a,r}+\eta^{a-1}R_2^{a-1}p^{a,r}(1-\phi^{a,r}_{P,pal}) \\
\dot{S_3^{a,r}} & = & \omega R_2^{a,r}+\omega R_3^{a,r}-\lambda_3^{a,r}(t)S_2^{a,r} & -\eta^a S_3^{a,r}+\eta^{a-1}S_3^{a-1}p^{a,r}(1-\phi^{a,r}_{P,pal}) \\
\dot{E_3^{a,r}} & = & \lambda_3^{a,r}(t)S_2^{a,r}-\sigma E_3^{a,r} & -\eta^a E_3^{a,r}+\eta^{a-1}E_3^{a-1}p^{a,r}(1-\phi^{a,r}_{P,pal}) \\
\dot{A_3^{a,r}} & = & p^a\sigma E_3^{a,r}-\gamma_3 A_3^{a,r}\rho & -\eta^a A_3^{a,r}+\eta^{a-1}A_3^{a-1}p^{a,r}(1-\phi^{a,r}_{P,pal}) \\
\dot{I_3^{a,r}} & = & (1-p^a)\sigma E_3^{a,r}-\gamma_3 I_3^{a,r} & -\eta^a I_3^{a,r}+\eta^{a-1}I_3^{a-1}p^{a,r}(1-\phi^{a,r}_{P,pal}) \\
\dot{R_3^{a,r}} & = & \rho\gamma_3 A_3^{a,r}+\gamma_3 I_3^{a,r}-\omega R_3^{a,r} & -\eta^a R_3^{a,r}+\eta^{a-1}R_3^{a-1}p^{a,r}(1-\phi^{a,r}_{P,pal}) \\
\dot{V_P^{a,r}} & = & \mu p^{a,r}\mathbb{1}_1(a)\phi^{a,r}_{P,pal}+(N^{a,r}-V_P^{a,r})\phi^{a,r}_{P,pal} & -\eta^a V_P^{a,r}+\eta^{a-1}V_P^{a-1}p^{a,r} \quad -\dot{V_P^{a,r}}\omega_{pal} \\
\dot{Z^{a,r}} & = & \sigma(E_0^{a,r}+E_1^{a,r}+E_2^{a,r}+E_3^{a,r}) &
\end{array} \tag{15}
$$

where an overdot refers to differentiation with respect to t, $\mathbb{1}_1(a)$ is the indicator function (non-zero at $a=1$), and the equation for the force of infection is:

$$
\lambda_i^{a,r}(t) = q_p f_i(t) \sum_{b=1}^{25} \frac{(\mathfrak{p}^{a,b}+q_c\mathfrak{c}^{a,b})}{N^b} \left(\sum_{r,\ i} A_i^{b,r}\alpha + I_i^{b,r} \right)
$$

where $\sum_{r,\ i}$ is the sum over all the Palivizumab eligible and non-Palivizumab eligible clinical-risk groups, and exposure groups $i=\{0,1,2,3\}$ and $f_i(t) = q_p(1+b_1\exp((t-\phi)^2/(2\psi^2))\prod_{i'=0}^{i}\delta_{i'}$. Further, $\phi^{a,r}_{P,pal}$ is the number of persons who are protected by Palivizumab in age group a and clinical-risk group r. The

initial conditions for this set of ODEs are given by **Equations 3** with $N = N^{a,r}$ and $V_P^{a,r} = 0$.

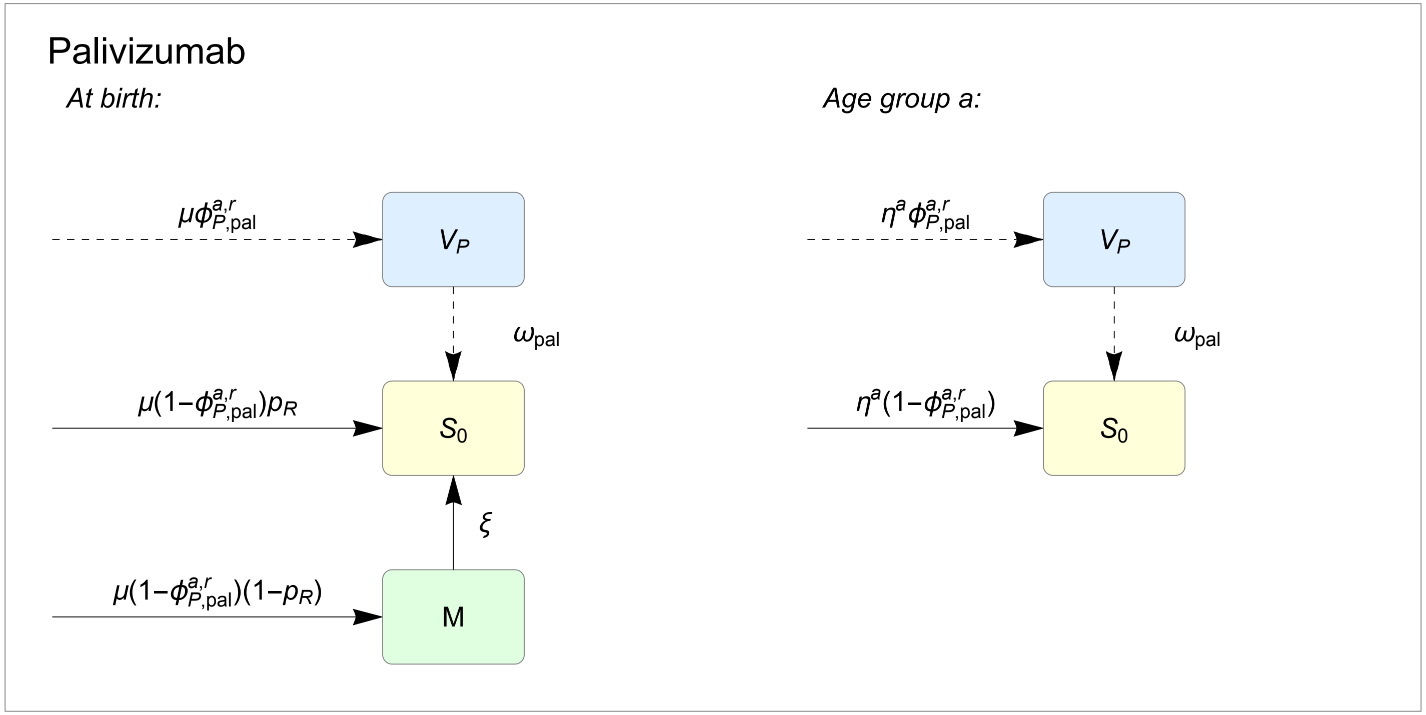

Figure 6: The relationship between the state variables (V_P: protected due to Palivizumab antibodies) used in the Palivizumab intervention model. For Palivizumab the parameters are μ, the birth rate, p_R the proportion of infants born with protection due to maternal immunity, and $\phi^{a,r}_{P,pal}$ the proportion of infants in age group a, clinical risk group r who are newly protected by Palivizumab at time t. The left schematic shows the rate of change between epidemiological groups when Palivizumab is administered at birth. The right schematic shows the rate of change between epidemiological groups when Palivizumab is given other age group. The rate of loss of immunity is given by ω_{pal} and shown by the dashed line. Rate of loss of maternal protection occurs at rate ξ and is shown by a solid line.

4.2 Long-acting monoclonal antibodies programmes

In order to evaluate to the impact of intervention programmes aimed at infants in different clinical risk groups, we stratified the infants into demographic groups according to their clinical-risk status i) Palivizumab-eligible (VHR), ii) high-risk (HR), and neither (NR) (indicated by the superscript r). To estimate the proportion of infants who are high-risk, we assume the prevalence is 3.8% across each monthly age group up to 11 months.[24]

The monoclonal antibody programmes considered are given below:

**Intervention programme name**	**Prophylactic(s)**	**Eligible population**	**Window of administration**	**Coverage of eligible population**	**Annual number of courses**	**Comparator**
MAB-VHR	La-mAB	VHR infants	October-February	90%	11,679	Palivizumab
MAB-HR-S	La-mAB	VHR infants	October-February	90%		MAB-VHR
	La-mAB	HR neonates	October-February	90%		
MAB-HR-S+	La-mAB	VHR infants	October-February	90%	22,907	MAB-VHR
	La-mAB	HR neonates	October-February	90%		
	La-mAB	HR 1-5 months	September-October	90%		
MAB-ALL-S	La-mAB	VHR infants	October-February	90%	252,581	MAB-HR-S+
	La-mAB	HR and HR neonates	October-February	90%		
MAB-ALL-S+	La-mAB	VHR infants	October-February	90%	547,818	MAB-ALL-S
	La-mAB	HR and HR neonates	October-February	90%		
	La-mAB	HR and HR 1-5 months	September-October	90%		

Table 5: Summary of the characteristics of the intervention programmes which use long-acting monoclonal antibodies. La-mAB: Long-acting monoclonal antibodies

The ODEs of the RSV intervention model for the long-acting monoclonal antibodies programmes, for age group a and clinical-risk group r are:

$$
\begin{array}{rcllr}
M^{\dot{a},r} & = & \overbrace{p_R \mu p^{a,r} \mathbb{1}_1(a)(1-\phi^{a,r}_{P,mab}) - \xi M^{a,r}}^{\text{Transmission terms}} & \overbrace{-\eta^a M^{a,r} + \eta^{a-1} M^{a-1} p^{a,r}(1-\phi^{a,r}_{P,mab})}^{\text{Ageing terms}} & \\
S_0^{\dot{a},r} & = & (1-p_R)\mu p^{a,r}\mathbb{1}_1(a)(1-\phi^{a,r}_{P,mab}) + \xi M^{a,s} - \lambda_0^{a,r}(t) S_0^{a,r} & -\eta^a S_0^{a,r} + \eta^{a-1} S_0^{a-1} p^{a,r}(1-\phi^{a,r}_{P,mab}) & \overbrace{+V_M^{\dot{a},r}\omega_{mab}}^{\text{Monoclonal antibodies terms}} \\
E_0^{\dot{a},r} & = & \lambda_0^{a,r}(t) S_0^{a,s} - \sigma E_0^{a,r} & -\eta^a E_0^{a,r} + \eta^{a-1} E_0^{a-1} p^{a,r}(1-\phi^{a,r}_{P,mab}) & \\
A_0^{\dot{a},r} & = & p^a \sigma E_0^{a,r} - \gamma_0 A_0^{a,r}\rho & -\eta^a A_0^{a,r} + \eta^{a-1} A_0^{a-1} p^{a,r}(1-\phi^{a,r}_{P,mab}) & \\
I_0^{\dot{a},r} & = & (1-p^a)\sigma E_0^{a,r} - \gamma_0 I_0^{a,r} & -\eta^a I_0^{a,r} + \eta^{a-1} I_0^{a-1} p^{a,r}(1-\phi^{a,r}_{P,mab}) & \\
R_0^{\dot{a},r} & = & \rho\gamma_0 A_0^{a,r} + \gamma_0 I_0^{a,r} - \omega R_0^{a,r} & -\eta^a R_0^{a,r} + \eta^{a-1} R_0^{a-1} p^{a,r}(1-\phi^{a,r}_{P,mab}) & \\
S_1^{\dot{a},r} & = & \omega R_0^{a,r} - \lambda_1^{a,r}(t) S_1^{a,r} & -\eta^a S_1^{a,r} + \eta^{a-1} S_1^{a-1} p^{a,r}(1-\phi^{a,r}_{P,mab}) & \\
E_1^{\dot{a},r} & = & \lambda_1^{a,r}(t) S_1^{a,r} - \sigma E_1^{a,r} & -\eta^a E_1^{a,r} + \eta^{a-1} E_1^{a-1} p^{a,r}(1-\phi^{a,r}_{P,mab}) & \\
A_1^{\dot{a},r} & = & p^a \sigma E_1^{a,r} - \gamma_1 A_1^{a,r}\rho & -\eta^a A_1^{a,r} + \eta^{a-1} A_1^{a-1} p^{a,r}(1-\phi^{a,r}_{P,mab}) & \\
I_1^{\dot{a},r} & = & (1-p^a)\sigma E_1^{a,r} - \gamma_1 I_1^{a,r} & -\eta^a I_1^{a,r} + \eta^{a-1} A_1^{a-1} p^{a,r}(1-\phi^{a,r}_{P,mab}) & \\
R_1^{\dot{a},r} & = & \rho\gamma_1 A_1^{a,r} + \gamma_1 I_1^{a,r} - \omega R_1^{a,r} & -\eta^a R_1^{a,r} + \eta^{a-1} R_1^{a-1} p^{a,r}(1-\phi^{a,r}_{P,mab}) & \\
S_2^{\dot{a},r} & = & \omega R_1^{a,r} - \lambda_2^{a,r}(t) S_2^{a,r} & -\eta^a S_2^{a,r} + \eta^{a-1} S_2^{a-1} p^{a,r}(1-\phi^{a,r}_{P,mab}) & \\
E_2^{\dot{a},r} & = & \lambda_2^{a,r}(t) S_2^{a,r} - \sigma E_2^{a,r} & -\eta^a E_2^{a,r} + \eta^{a-1} E_2^{a-1} p^{a,r}(1-\phi^{a,r}_{P,mab}) & \\
A_2^{\dot{a},r} & = & p^a \sigma E_2^{a,r} - \gamma_2 A_2^{a,r}\rho & -\eta^a A_2^{a,r} + \eta^{a-1} A_2^{a-1} p^{a,r}(1-\phi^{a,r}_{P,mab}) & \\
I_2^{\dot{a},r} & = & (1-p^a)\sigma E_2^{a,r} - \gamma_2 I_2^{a,r} & -\eta^a I_2^{a,r} + \eta^{a-1} I_2^{a-1} p^{a,r}(1-\phi^{a,r}_{P,mab}) & \\
R_2^{\dot{a},r} & = & \rho\gamma_2 A_2^{a,r} + \gamma_2 I_2^{a,r} - \omega R_2^{a,r} & -\eta^a R_2^{a,r} + \eta^{a-1} R_2^{a-1} p^{a,r}(1-\phi^{a,r}_{P,mab}) & \\
S_3^{\dot{a},r} & = & \omega R_2^{a,r} + \omega R_3^{a,r} - \lambda_3^{a,r}(t) S_2^{a,r} & -\eta^a S_3^{a,r} + \eta^{a-1} S_3^{a-1} p^{a,r}(1-\phi^{a,r}_{P,mab}) & \\
E_3^{\dot{a},r} & = & \lambda_3^{a,r}(t) S_2^{a,r} - \sigma E_3^{a,r} & -\eta^a E_3^{a,r} + \eta^{a-1} E_3^{a-1} p^{a,r}(1-\phi^{a,r}_{P,mab}) & \\
A_3^{\dot{a},r} & = & p^a \sigma E_3^{a,r} - \gamma_3 A_3^{a,r}\rho & -\eta^a A_3^{a,r} + \eta^{a-1} A_3^{a-1} p^{a,r}(1-\phi^{a,r}_{P,mab}) & \\
I_3^{\dot{a},r} & = & (1-p^a)\sigma E_3^{a,r} - \gamma_3 I_3^{a,r} & -\eta^a I_3^{a,r} + \eta^{a-1} I_3^{a-1} p^{a,r}(1-\phi^{a,r}_{P,mab}) & \\
R_3^{\dot{a},r} & = & \rho\gamma_3 A_3^{a,r} + \gamma_3 I_3^{a,r} - \omega R_3^{a,r} & -\eta^a R_3^{a,r} + \eta^{a-1} R_3^{a-1} p^{a,r}(1-\phi^{a,r}_{P,mab}) & \\
V_M^{\dot{a},r} & = & \mu p^{a,r}\mathbb{1}_1(a)\phi^{a,r}_{P,mab} + (N^{a,r} - V_P^{a,r})\phi^{a,r}_{P,mab} & -\eta^a V_M^{a,r} + \eta^{a-1} V_M^{a-1} p^{a,r} & -V_M^{\dot{a},r}\omega_{mab} \\
Z^{\dot{a},r} & = & \sigma(E_0^{a,r} + E_1^{a,r} + E_2^{a,r} + E_3^{a,r}) & &
\end{array} \tag{16}
$$

where an overdot refers to differentiation with respect to t, $\mathbb{1}_1(a)$ is the indicator function (non-zero at $a = 1$), and the equations for the force of infection is:

$$
\lambda_i^{a,r}(t) = q_p f_i(t) \sum_{b=1}^{25} \frac{(\mathfrak{p}^{a,b} + q_c \mathfrak{c}^{a,b})}{N^b} \left(\sum_{r,\, i} A_i^{b,r}\alpha + I_i^{b,r} \right)
$$

where $\sum_{r,\, i}$ is the sum over all risk groups $\mathcal{R} = \{NR, HR, VHR\}$ and exposure groups $i = \{0, 1, 2, 3\}$ and $f_i(t) = q_p(1 + b_1 \exp((t-\phi)^2/(2\psi^2)) \prod_{i'=0}^{i} \delta_{i'}$. Further, $\phi^{a,r}_{P,mab}$ is the number of persons who are protected by monoclonal antibodies in age group a and clinical risk group r,.

The initial conditions for this set of ODEs are given by **Equations (3)** with $N = N^{a,r}$ and $V_M^{a,r} = 0$.

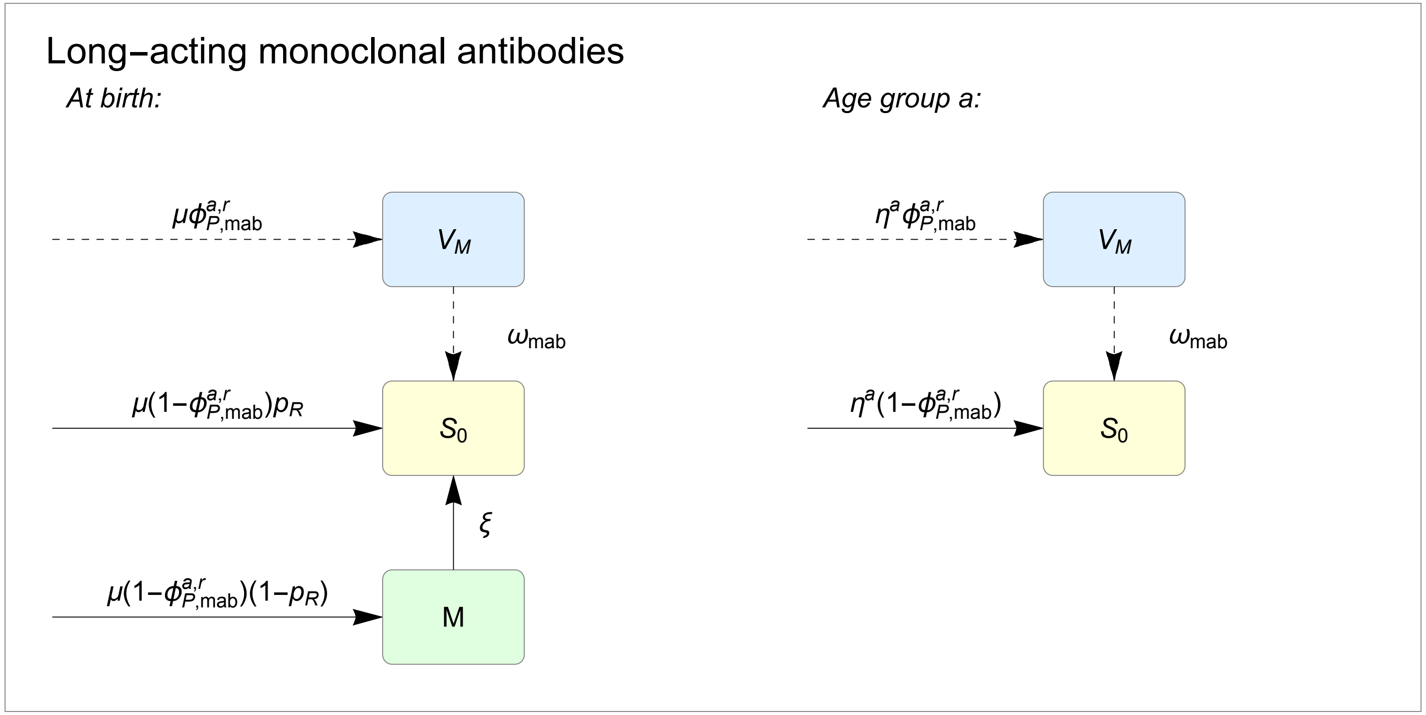

Figure 7: The relationship between the state variables (V_M: protected due to long-acting monoclonal antibodies) used in the long-acting monoclonal antibodies intervention model. For long-acting monoclonal antibodies the parameters are μ, the birth rate, p_R the proportion of infants born with protection due to maternal immunity, and $\phi^{a,r}_{P,mab}$ the proportion of infants in age group a, clinical risk group r who are newly protected by long-acting monoclonal antibodies at time t. The left schematic shows the rate of change between epidemiological groups when monoclonal antibodies are administered at birth. The right schematic shows the rate of change between epidemiological groups when long-acting monoclonal antibodies are given other age group. The rate of loss of immunity is given by ω_{mab} and shown by the dashed line. Rate of loss of maternal protection occurs at rate ξ and is shown by a solid line.

4.3 Childhood/elderly vaccination programmes

The childhood and elderly intervention programmes considered are given below:

**Intervention programme name**	**Prophylactic(s)**	**Eligible population**	**Window of administration**	**Coverage of eligible population**	**Annual number of courses**	**Comparator**
VAC-INF-S	Palivizumab	VHR infants	October-February	90%	2,128	Palivizumab
	Vaccine	2-month-olds	September-January	90%	251,162	
VAC-INF-A	Palivizumab	VHR infants	October-February	90%	2,128	VAC-INF-S
	Vaccine	2-month-olds	Year-round	90%	617,724	
VAC-2–4	Palivizumab	VHR infants	October-February	90%	2,128	VAC-INF-A
	Vaccine	2–4 year olds	October-February	45%	917,008	
VAC-5–9	Palivizumab	VHR infants	October-February	90%	2,128	VAC-2–4
	Vaccine	5–9 year olds	October-February	60%	2,046,820	
VAC-5–14	Palivizumab	VHR infants	October-February	90%	2,128	VAC-5–9
	Vaccine	5–14 year olds	August-December	60%	4,093,640	
VAC-75+	Palivizumab	VHR infants	October-February	90%	2,128	VAC-5–14
	Vaccine	75+ year olds	November-March	70%	5,495,680	
VAC-65+	Palivizumab	VHR infants	October-February	90%	2,128	VAC-75+
	Vaccine	65+ year olds	November-March	70%	10,281,800	

Table 6: Summary of the characteristics of the intervention programmes which use vaccines

The ODEs of the RSV intervention model for the above childhood and elderly programmes, for age group a and clinical-risk group r are:

$$
\begin{array}{rcllll}
 & & \overbrace{\qquad\qquad\qquad\qquad\qquad\qquad}^{\text{Transmission terms}} & \overbrace{\qquad\qquad\qquad\qquad\qquad\qquad}^{\text{Ageing terms}} & & \\
\dot{M}^{a,r} & = & p_R \mu p^{a,r} \mathbb{1}_1(a)(1-\phi^{a,r}_{P,pal}) - \xi M^{a,r} & -\eta^a M^{a,r} + \eta^{a-1} M^{a-1} p^{a,r}(1-\phi^{a,r}_{P,pal}) & & \\
 & & & & \overbrace{\qquad}^{\textbf{Palivizumab terms}} & \overbrace{\qquad}^{\text{Vaccination terms}} \\
\dot{S}_0^{a,r} & = & (1-p_R)\mu p^{a,r} \mathbb{1}_1(a)(1-\phi^{a,r}_{P,pal}) + \xi M^{a,s} - \lambda_0^{a,r}(t) S_0^{a,r} & -\eta^a S_0^{a,r} + \eta^{a-1} S_0^{a-1} p^{a,r}(1-\phi^{a,r}_{P,pal}) & +\dot{V}_P^{a,r}\omega_{pal} & -\bar{S}_0^{a,r}\phi^{a,r}_{P,vac} \\
\dot{E}_0^{a,r} & = & \lambda_0^{a,r}(t) S_0^{a,s} - \sigma E_0^{a,r} & -\eta^a E_0^{a,r} + \eta^{a-1} E_0^{a-1} p^{a,r}(1-\phi^{a,r}_{P,pal}) & & \\
\dot{A}_0^{a,r} & = & p^a \sigma E_0^{a,r} - \gamma_0 A_0^{a,r} \rho & -\eta^a A_0^{a,r} + \eta^{a-1} A_0^{a-1} p^{a,r}(1-\phi^{a,r}_{P,pal} & & \\
\dot{I}_0^{a,r} & = & (1-p^a)\sigma E_0^{a,r} - \gamma_0 I_0^{a,r} & -\eta^a I_0^{a,r} + \eta^{a-1} I_0^{a-1} p^{a,r}(1-\phi^{a,r}_{P,pal}) & & \\
\dot{R}_0^{a,r} & = & \rho\gamma_0 A_0^{a,r} + \gamma_0 I_0^{a,r} - \omega R_0^{a,r} & -\eta^a R_0^{a,r} + \eta^{a-1} R_0^{a-1} p^{a,r}(1-\phi^{a,r}_{P,pal}) & & +\bar{S}_0^{a,r}\phi^{a,r}_{P,vac} \\
\dot{S}_1^{a,r} & = & \omega R_0^{a,r} - \lambda_1^{a,r}(t) S_1^{a,r} & -\eta^a S_1^{a,r} + \eta^{a-1} S_1^{a-1} p^{a,r}(1-\phi^{a,r}_{P,pal}) & & -\bar{S}_1^{a,r}\phi^{a,r}_{P,vac} \\
\dot{E}_1^{a,r} & = & \lambda_1^{a,r}(t) S_1^{a,r} - \sigma E_1^{a,r} & -\eta^a E_1^{a,r} + \eta^{a-1} E_1^{a-1} p^{a,r}(1-\phi^{a,r}_{P,pal}) & & \\
\dot{A}_1^{a,r} & = & p^a \sigma E_1^{a,r} - \gamma_1 A_1^{a,r} \rho & -\eta^a A_1^{a,r} + \eta^{a-1} A_1^{a-1} p^{a,r}(1-\phi^{a,r}_{P,pal}) & & \\
\dot{I}_1^{a,r} & = & (1-p^a)\sigma E_1^{a,r} - \gamma_1 I_1^{a,r} & -\eta^a I_1^{a,r} + \eta^{a-1} A_1^{a-1} p^{a,r}(1-\phi^{a,r}_{P,pal}) & & \\
\dot{R}_1^{a,r} & = & \rho\gamma_1 A_1^{a,r} + \gamma_1 I_1^{a,r} - \omega R_1^{a,r} & -\eta^a R_1^{a,r} + \eta^{a-1} R_1^{a-1} p^{a,r}(1-\phi^{a,r}_{P,pal}) & & +\bar{S}_1^{a,r}\phi^{a,r}_{P,vac} \\
\dot{S}_2^{a,r} & = & \omega R_1^{a,r} - \lambda_2^{a,r}(t) S_2^{a,r} & -\eta^a S_2^{a,r} + \eta^{a-1} S_2^{a-1} p^{a,r}(1-\phi^{a,r}_{P,pal}) & & -\bar{S}_2^{a,r}\phi^{a,r}_{P,vac} \\
\dot{E}_2^{a,r} & = & \lambda_2^{a,r}(t) S_2^{a,r} - \sigma E_2^{a,r} & -\eta^a E_2^{a,r} + \eta^{a-1} E_2^{a-1} p^{a,r}(1-\phi^{a,r}_{P,pal}) & & \\
\dot{A}_2^{a,r} & = & p^a \sigma E_2^{a,r} - \gamma_2 A_2^{a,r} \rho & -\eta^a A_2^{a,r} + \eta^{a-1} A_2^{a-1} p^{a,r}(1-\phi^{a,r}_{P,pal}) & & \\
\dot{I}_2^{a,r} & = & (1-p^a)\sigma E_2^{a,r} - \gamma_2 I_2^{a,r} & -\eta^a I_2^{a,r} + \eta^{a-1} I_2^{a-1} p^{a,r}(1-\phi^{a,r}_{P,pal}) & & \\
\dot{R}_2^{a,r} & = & \rho\gamma_2 A_2^{a,r} + \gamma_2 I_2^{a,r} - \omega R_2^{a,r} & -\eta^a R_2^{a,r} + \eta^{a-1} R_2^{a-1} p^{a,r}(1-\phi^{a,r}_{P,pal}) & & +\bar{S}_2^{a,r}\phi^{a,r}_{P,vac} \\
\dot{S}_3^{a,r} & = & \omega R_2^{a,r} + \omega R_3^{a,r} - \lambda_3^{a,r}(t) S_2^{a,r} & -\eta^a S_3^{a,r} + \eta^{a-1} S_3^{a-1} p^{a,r}(1-\phi^{a,r}_{P,pal}) & & -\bar{S}_3^{a,r}\phi^{a,r}_{P,vac} \\
\dot{E}_3^{a,r} & = & \lambda_3^{a,r}(t) S_2^{a,r} - \sigma E_3^{a,r} & -\eta^a E_3^{a,r} + \eta^{a-1} E_3^{a-1} p^{a,r}(1-\phi^{a,r}_{P,pal}) & & \\
\dot{A}_3^{a,r} & = & p^a \sigma E_3^{a,r} - \gamma_3 A_3^{a,r} \rho & -\eta^a A_3^{a,r} + \eta^{a-1} A_3^{a-1} p^{a,r}(1-\phi^{a,r}_{P,pal}) & & \\
\dot{I}_3^{a,r} & = & (1-p^a)\sigma E_3^{a,r} - \gamma_3 I_3^{a,r} & -\eta^a I_3^{a,r} + \eta^{a-1} I_3^{a-1} p^{a,r}(1-\phi^{a,r}_{P,pal}) & & \\
\dot{R}_3^{a,r} & = & \rho\gamma_3 A_3^{a,r} + \gamma_3 I_3^{a,r} - \omega R_3^{a,r} & -\eta^a R_3^{a,r} + \eta^{a-1} R_3^{a-1} p^{a,r}(1-\phi^{a,r}_{P,pal}) & & +\bar{S}_3^{a,r}\phi^{a,r}_{P,vac} \\
\dot{V}_P^{a,r} & = & \mu p^{a,r} \mathbb{1}_1(a)\phi^{a,r}_{P,pal} + (N^{a,r} - V_P^{a,r})\phi^{a,r}_{P,pal} & -\eta^a V_P^{a,r} + \eta^{a-1} V_P^{a-1} p^{a,r} & -\dot{V}_P^{a,r}\omega_{pal} & \\
\dot{Z}^{a,r} & = & \sigma(E_0^{a,r} + E_1^{a,r} + E_2^{a,r} + E_3^{a,r}) & & &
\end{array}
\tag{17}
$$

where an overdot refers to differentiation with respect to t, $\mathbb{1}_1(a)$ is the indicator function (non-zero at $a = 1$), and the equation for the force of infection is:

$$\lambda_i^{a,r}(t) = q_p f_i(t) \sum_{b=1}^{25} \frac{(\mathfrak{p}^{a,b} + q_c \mathfrak{c}^{a,b})}{N^b} \left(\sum_{r,\ i} A_i^{b,r}\alpha + I_i^{b,r} \right)$$

where $\sum_{r,\ i}$ is the sum over all risk groups $\mathcal{R} = \{NR, HR, VHR\}$ and exposure groups $i = \{0,1,2,3\}$ and $f_i(t) = q_p(1 + b_1 \exp((t-\phi)^2/(2\psi^2))\prod_{i'=0}^{i}\delta_{i'}$. Further, $\phi^{a,r}_{P,pal}$ is the number of persons who are protected by Palivizumab in age group a and clinical risk group r, and $\phi^{a,r}_{P,vac}$ is the number of persons protected

by vaccination in age group a and clinical risk group a at time t.

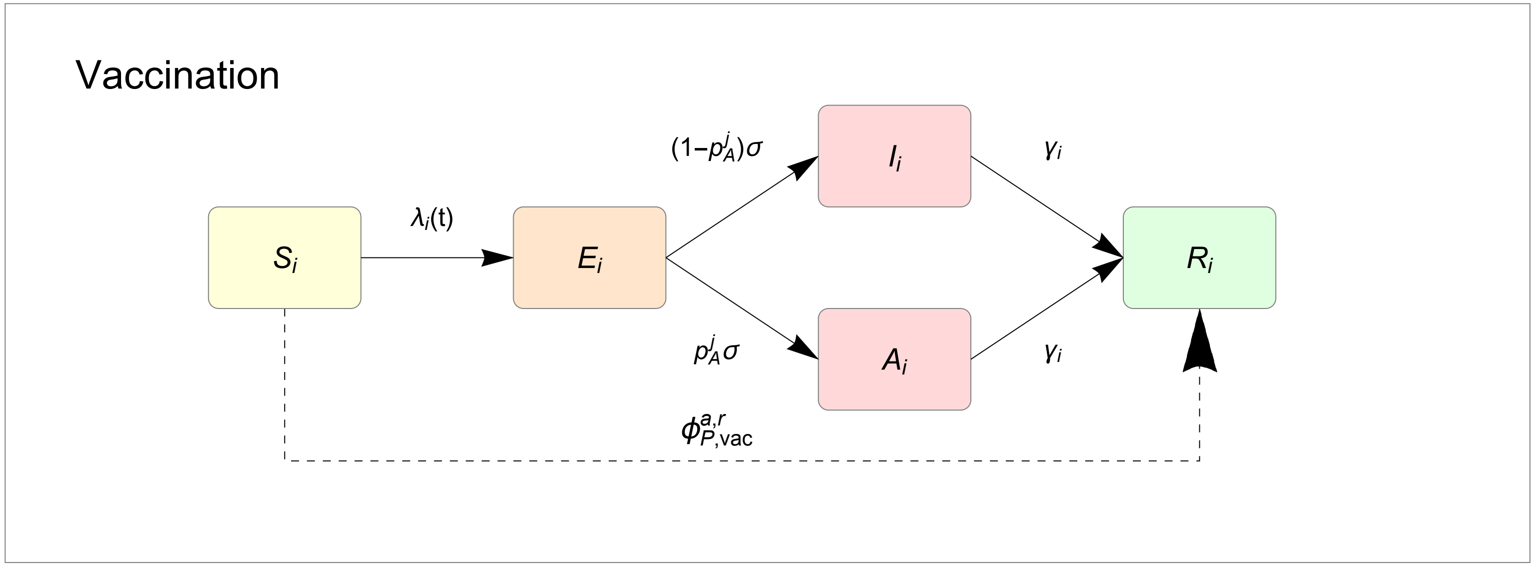

Figure 8: The relationship between state variables for vaccination in children or the elderly. Here $\phi_{P,vac}^{a,r}$ is the proportion of individuals in age group a, clinical risk group r who are newly protected by vaccination at time t. Solid lines refer to natural disease progression and dashed lines refer to immune progression due to vaccination.

The initial conditions for this set of ODEs are given by **Equations (3)** with $N = N^{a,r}$ and $V_P^{a,r} = 0$.

4.4 Maternal vaccine programmes

The proportion of persons in age group a who are mothers with an infant less than 1 years of age, u^a, was calculated by multiplying the total number of infants less than 1 by the age-specific proportion of births by parental age u_p^a (u_p^a is non-zero for $a = 19, 20$ and 21 only).[25] This gives proportions of 0.0175, 0.0601, and 0.0233 for 15–24, 25–34, and 35–44 years respectively.[25]The proportion of mothers who are in the programme is given by $\phi_c = 0.6$. We define, $u^{a,p} = u^a(1 - \phi_c)$, $u^{a,c} = u^a\phi_c$ and $u^{a,n} = (1 - u^a)$.

When evaluating the maternal vaccination programmes, the age and clinical-risk groups are further stratified according to maternal status and whether they are including in the programme (see **Table 7**). The strategy is the same as outlined in a previous mathematical model which evaluates the impact the maternal Pertussis vaccines.[26]

**Superscript**	**Description**
n	Infants less than 1 years of age and who are not participating in the maternal vaccination programmes and adults who are not mothers who have given birth in the last year
p	Mothers who have given birth in the last year who are not in the maternal vaccine programme
c	Mothers who have given birth in the last year and are in the maternal vaccine programme and the newly born infant.

Table 7: Summary of the maternal vaccine-related states.

The maternal vaccine programmes considered are given below:

**Intervention programme name**	**Prophylactic(s)**	**Eligible population**	**Window of administration**	**Coverage of eligible population**	**Annual number of courses**	**Comparator**
MAT-S	Palivizumab	VHR infants	August-December	90%	2,128	Palivizumab
	Maternal vaccine	Pregnant women 28-32 weeks gestational age	October-February	60%	165,257	
MAT-A	Palivizumab	VHR infants	October-February	90%	2,128	MAT-S
	Maternal vaccine	Pregnant women 28-32 weeks gestational age	Year-round	60%	406,442	

Table 8: Summary of the characteristics of the intervention programmes which use maternal-vaccines

$$
\begin{array}{rclllll}
 & & \overbrace{\hphantom{xxxxxxxxxxxxxxxxxxxxxxxxxxxxxxxxxxxxxxxxxxxxxxxxxxxxx}}^{\text{Transmission terms}} & \overbrace{\hphantom{xxxxxxxxxxxxxxxxxxxxxxxxxxxxxx}}^{\text{Ageing terms}} & & \\
\dot{M^{a,r,s}} & = & p_R\mu p^{a,r,s}u^{a,s}\mathbb{1}_1(a)(1-\phi^{a,r,s}_{P,pal})(1-\phi^{a,r,s}_{P,mat}) + \phi^{a,r,s}_{P,mat}\mu p^{a,r,s}u^{a,s}\mathbb{1}_1(a) - \xi M^{a,r,s} & -\eta^a M^{a,r,s} + \eta^{a-1}M^{a-1}p^{a,r,s}u^a\phi_c(1-\phi^{a,r,s}_{P,pal}) & & \\
 & & & & \overbrace{\hphantom{xxxxxxxx}}^{\text{Palivizumab terms}} & \overbrace{\hphantom{xxxxxxxx}}^{\text{Vaccination terms}} \\
\dot{S_0^{a,r,s}} & = & (1-p_R)\mu p^{a,r,s}u^{a,s}\mathbb{1}_1(a)(1-\phi^{a,r,s}_{P,pal}) + \xi M^{a,s} - \lambda_0^{a,r,s}(t)S_0^{a,r,s} & -\eta^a S_0^{a,r,s} + \eta^{a-1}S_0^{a-1}p^{a,r,s}u^{a,s}(1-\phi^{a,r,s}_{P,pal}) & +\dot{V_P^{a,r,s}}\omega_{pal} & -\bar{S}_0^{a,r,s}\phi^{a,r,s}_{P,mat} \\
\dot{E_0^{a,r,s}} & = & \lambda_0^{a,r,s}(t)S_0^{a,s} - \sigma E_0^{a,r,s} & -\eta^a E_0^{a,r,s} + \eta^{a-1}E_0^{a-1}p^{a,r,s}u^{a,s}(1-\phi^{a,r,s}_{P,pal}) & & \\
\dot{A_0^{a,r,s}} & = & p^a\sigma E_0^{a,r,s} - \gamma_0 A_0^{a,r,s}\rho & -\eta^a A_0^{a,r,s} + \eta^{a-1}A_0^{a-1}p^{a,r,s}u^{a,s}(1-\phi^{a,r,s}_{P,pal} & & \\
\dot{I_0^{a,r,s}} & = & (1-p^a)\sigma E_0^{a,r,s} - \gamma_0 I_0^{a,r,s} & -\eta^a I_0^{a,r,s} + \eta^{a-1}I_0^{a-1}p^{a,r,s}u^{a,s}(1-\phi^{a,r,s}_{P,pal}) & & \\
\dot{R_0^{a,r,s}} & = & \rho\gamma_0 A_0^{a,r,s} + \gamma_0 I_0^{a,r,s} - \omega R_0^{a,r,s} & -\eta^a R_0^{a,r,s} + \eta^{a-1}R_0^{a-1}p^{a,r,s}u^{a,s}(1-\phi^{a,r,s}_{P,pal}) & & +\bar{S}_0^{a,r,s}\phi^{a,r,s}_{P,mat} \\
\dot{S_1^{a,r,s}} & = & \omega R_0^{a,r,s} - \lambda_1^{a,r,s}(t)S_1^{a,r,s} & -\eta^a S_1^{a,r,s} + \eta^{a-1}S_1^{a-1}p^{a,r,s}u^{a,s}(1-\phi^{a,r,s}_{P,pal}) & & -\bar{S}_1^{a,r,s}\phi^{a,r,s}_{P,mat} \\
\dot{E_1^{a,r,s}} & = & \lambda_1^{a,r,s}(t)S_1^{a,r,s} - \sigma E_1^{a,r,s} & -\eta^a E_1^{a,r,s} + \eta^{a-1}E_1^{a-1}p^{a,r,s}u^{a,s}(1-\phi^{a,r,s}_{P,pal}) & & \\
\dot{A_1^{a,r,s}} & = & p^a\sigma E_1^{a,r,s} - \gamma_1 A_1^{a,r,s}\rho & -\eta^a A_1^{a,r,s} + \eta^{a-1}A_1^{a-1}p^{a,r,s}u^{a,s}(1-\phi^{a,r,s}_{P,pal}) & & \\
\dot{I_1^{a,r,s}} & = & (1-p^a)\sigma E_1^{a,r,s} - \gamma_1 I_1^{a,r,s} & -\eta^a I_1^{a,r,s} + \eta^{a-1}A_1^{a-1}p^{a,r,s}u^{a,s}(1-\phi^{a,r,s}_{P,pal}) & & \\
\dot{R_1^{a,r,s}} & = & \rho\gamma_1 A_1^{a,r,s} + \gamma_1 I_1^{a,r,s} - \omega R_1^{a,r,s} & -\eta^a R_1^{a,r,s} + \eta^{a-1}R_1^{a-1}p^{a,r,s}u^{a,s}(1-\phi^{a,r,s}_{P,pal}) & & +\bar{S}_1^{a,r,s}\phi^{a,r,s}_{P,mat} \\
\dot{S_2^{a,r,s}} & = & \omega R_1^{a,r,s} - \lambda_2^{a,r,s}(t)S_2^{a,r,s} & -\eta^a S_2^{a,r,s} + \eta^{a-1}S_2^{a-1}p^{a,r,s}u^{a,s}(1-\phi^{a,r,s}_{P,pal}) & & -\bar{S}_2^{a,r,s}\phi^{a,r,s}_{P,mat} \\
\dot{E_2^{a,r,s}} & = & \lambda_2^{a,r,s}(t)S_2^{a,r,s} - \sigma E_2^{a,r,s} & -\eta^a E_2^{a,r,s} + \eta^{a-1}E_2^{a-1}p^{a,r,s}u^{a,s}(1-\phi^{a,r,s}_{P,pal}) & & \\
\dot{A_2^{a,r,s}} & = & p^a\sigma E_2^{a,r,s} - \gamma_2 A_2^{a,r,s}\rho & -\eta^a A_2^{a,r,s} + \eta^{a-1}A_2^{a-1}p^{a,r,s}u^{a,s}(1-\phi^{a,r,s}_{P,pal}) & & \\
\dot{I_2^{a,r,s}} & = & (1-p^a)\sigma E_2^{a,r,s} - \gamma_2 I_2^{a,r,s} & -\eta^a I_2^{a,r,s} + \eta^{a-1}I_2^{a-1}p^{a,r,s}u^{a,s}(1-\phi^{a,r,s}_{P,pal}) & & \\
\dot{R_2^{a,r,s}} & = & \rho\gamma_2 A_2^{a,r,s} + \gamma_2 I_2^{a,r,s} - \omega R_2^{a,r,s} & -\eta^a R_2^{a,r,s} + \eta^{a-1}R_2^{a-1}p^{a,r,s}u^{a,s}(1-\phi^{a,r,s}_{P,pal}) & & +\bar{S}_2^{a,r,s}\phi^{a,r,s}_{P,mat} \\
\dot{S_3^{a,r,s}} & = & \omega R_2^{a,r,s} + \omega R_3^{a,r,s} - \lambda_3^{a,r,s}(t)S_2^{a,r,s} & -\eta^a S_3^{a,r,s} + \eta^{a-1}S_3^{a-1}p^{a,r,s}u^{a,s}(1-\phi^{a,r,s}_{P,pal}) & & -\bar{S}_3^{a,r,s}\phi^{a,r,s}_{P,mat} \\
\dot{E_3^{a,r,s}} & = & \lambda_3^{a,r,s}(t)S_2^{a,r,s} - \sigma E_3^{a,r,s} & -\eta^a E_3^{a,r,s} + \eta^{a-1}E_3^{a-1}p^{a,r,s}u^{a,s}(1-\phi^{a,r,s}_{P,pal}) & & \\
\dot{A_3^{a,r,s}} & = & p^a\sigma E_3^{a,r,s} - \gamma_3 A_3^{a,r,s}\rho & -\eta^a A_3^{a,r,s} + \eta^{a-1}A_3^{a-1}p^{a,r,s}u^{a,s}(1-\phi^{a,r,s}_{P,pal}) & & \\
\dot{I_3^{a,r,s}} & = & (1-p^a)\sigma E_3^{a,r,s} - \gamma_3 I_3^{a,r,s} & -\eta^a I_3^{a,r,s} + \eta^{a-1}I_3^{a-1}p^{a,r,s}u^{a,s}(1-\phi^{a,r,s}_{P,pal}) & & \\
\dot{R_3^{a,r,s}} & = & \rho\gamma_3 A_3^{a,r,s} + \gamma_3 I_3^{a,r,s} - \omega R_3^{a,r,s} & -\eta^a R_3^{a,r,s} + \eta^{a-1}R_3^{a-1}p^{a,r,s}u^{a,s}(1-\phi^{a,r,s}_{P,pal}) & & +\bar{S}_3^{a,r,s}\phi^{a,r,s}_{P,mat} \\
\dot{V_P^{a,r,s}} & = & \mu p^{a,r,s}u^{a,s}\mathbb{1}_1(a)\phi^{a,r,s}_{P,pal} + (N^{a,r,s} - V_P^{a,r,s})\phi^{a,r,s}_{P,pal} & -\eta^a V_P^{a,r,s} + \eta^{a-1}V_P^{a-1}p^{a,r}u^{a,s} & -\dot{V_P^{a,r,s}}\omega_{pal} & \\
\dot{Z^{a,r,s}} & = & \sigma(E_0^{a,r,s} + E_1^{a,r,s} + E_2^{a,r,s} + E_3^{a,r,s}) & & &
\end{array}
\tag{18}
$$

where the force of infection is given by defining $\mathcal{I}^{b,s} = \sum_{i,r} A_i^{b,r,s}\alpha + I_i^{b,r,s}$ for maternal vaccine groups, $s = \{n, p, c\}$, then the equations for the force of infection for the three maternal vaccine states are:

$$
\lambda_i^{a,r,n}(t) = q_p f_i(t)\sum_{b=1}^{25}\left[\frac{(\mathfrak{p}^{a(n),b(n)} + q_c\mathfrak{c}^{a(n),b(n)})}{N^{b,n}}\mathcal{I}^{b,n} + \frac{\mathfrak{p}^{a(n),b(p)} + q_c\mathfrak{c}^{a(n),b(p)}}{N^{b,p}}\mathcal{I}^{b,p} + \frac{\mathfrak{p}^{a(n),b(c)} + q_c\mathfrak{c}^{a(n),b(c)}}{N^{b,c}}\mathcal{I}^{b,c}\right] \tag{19}
$$

$$\lambda_i^{a,r,p}(t) = q_p f_i(t) \sum_{b=1}^{25} \left[\frac{(\mathfrak{p}^{a(p),b(n)} + q_c \mathfrak{c}^{a(p),b(n)})}{N^{b,n}} \mathcal{I}^{b,n} + \frac{\mathfrak{p}^{a(p),b(p)} + q_c \mathfrak{c}^{a(p),b(p)}}{N^{b,p}} \mathcal{I}^{b,p} + \frac{\mathfrak{p}^{a(p),b(c)} + q_c \mathfrak{c}^{a(p),b(c)}}{N^{b,c}} \mathcal{I}^{b,c} \right] \tag{20}$$

$$\lambda_i^{a,r,c}(t) = q_p f_i(t) \sum_{b=1}^{25} \left[\frac{(\mathfrak{p}^{a(c),b(n)} + q_c \mathfrak{c}^{a(c),b(n)})}{N^{b,n}} \mathcal{I}^{b,n} + \frac{\mathfrak{p}^{a(c),b(p)} + q_c \mathfrak{c}^{a(c),b(p)}}{N^{b,p}} \mathcal{I}^{b,p} + \frac{\mathfrak{p}^{a(c),b(c)} + q_c \mathfrak{c}^{a(c),b(c)}}{N^{b,c}} \mathcal{I}^{b,c} \right] \tag{21}$$

where the contact matrices are defined in **Table 9**. These contact matrices are modified versions of the matrices outlined in the mathematical model used to evaluate the impact of maternal Pertussis vaccines.[26]

**Participant**		**Contact**, Age group (a), Maternal-vaccine group (s_2)					
		<1yrs		15–44yrs			1-14,45+
Age group (a) (yrs)	Maternal-vaccine group (s_1)	n	c	n	c	p	n
<1	n	$\mathfrak{p}^{a,b}(1-\phi_c)$	$\mathfrak{p}^{a,b}\phi_c$	$\frac{\mathfrak{p}_H^{a,b}}{2} + (\mathfrak{p}^{a,b} - \mathfrak{p}_H^{a,b})(1-u^b)$	$(\mathfrak{p}^{a,b} - \mathfrak{p}_H^{a,b})u^b\phi_c$	$\frac{\mathfrak{p}_H^{a,b}}{2} + (\mathfrak{p}^{a,b} - \mathfrak{p}_H^{a,b})u^b(1-\phi_c)$	$\mathfrak{p}^{a,b}$
	c	$\mathfrak{p}^{a,b}(1-\phi_c)$	$\mathfrak{p}^{a,b}\phi_c$	$\frac{\mathfrak{p}_H^{a,b}}{2} + (\mathfrak{p}^{a,b} - \mathfrak{p}_H^{a,b})(1-u^b)$	$\frac{\mathfrak{p}_H^{a,b}}{2} + (\mathfrak{p}^{a,b} - \mathfrak{p}_H^{a,b})u^b\phi_c$	$(\mathfrak{p}^{a,b} - \mathfrak{p}_H^{a,b})u^b(1-\phi_c)$	$\mathfrak{p}^{a,b}$
15–44	n	$\mathfrak{p}^{a,b}(1-\phi_c)$	$\mathfrak{p}^{a,b}\phi_c$	$\mathfrak{p}^{a,b}(1-u^b)$	$\mathfrak{p}^{a,b}u^b\phi_c$	$\mathfrak{p}^{a,b}u^b(1-\phi_c)$	$\mathfrak{p}^{a,b}$
	c	$(\mathfrak{p}^{a,b} - \mathfrak{p}_H^{a,b})(1-\phi_c)$	$\mathfrak{p}_H^{a,b} + (\mathfrak{p}^{a,b} - \mathfrak{p}_H^{a,b})\phi_c$	$\mathfrak{p}^{a,b}(1-u^b)$	$\mathfrak{p}^{a,b}u^b\phi_c$	$\mathfrak{p}^{a,b}u^b(1-\phi_c)$	$\mathfrak{p}^{a,b}$
	p	$(\mathfrak{p}^{a,b} - \mathfrak{p}_H^{a,b})(1-\phi_c)$	$\mathfrak{p}_H^{a,b} + (\mathfrak{p}^{a,b} - \mathfrak{p}_H^{a,b})\phi_c$	$\mathfrak{p}^{a,b}(1-u^b)$	$\mathfrak{p}^{a,b}u^b\phi_c$	$\mathfrak{p}^{a,b}u^b(1-\phi_c)$	$\mathfrak{p}^{a,b}$
1–14, 45+	n	$\mathfrak{p}^{a,b}(1-\phi_c)$	$\mathfrak{p}^{a,b}\phi_c$	$\mathfrak{p}^{a,b}(1-u^b)$	$\mathfrak{p}^{a,b}u^b\phi_c$	$\mathfrak{p}^{a,b}u^b(1-\phi_c)$	$\mathfrak{p}^{a,b}$

Table 9: Formulae for synthesing the contact matrices with maternal-vaccine stratification.

Symbol	Definition	Source
$\mathfrak{p}_H^{a,b}$	Number of daily household physical contacts only made by age group a with age group b	[3,4]
$\mathfrak{p}^{a(s_1),b(s_2)}$	Total number of daily household physical contacts made by age group a and maternal vaccine group s_1 with age group b and maternal vaccine group s_2. $(s_i = \{n, p, c\})$	Generated by **Table 9**
$\mathfrak{c}_H^{a,b}$	Number of daily household conversational contacts only made by age group a with age group b	[3,4]
$\mathfrak{c}^{a(s_1),b(s_2)}$	Total number of daily conversational contacts made by age group a and maternal vaccine group s_1 with age group b and maternal vaccine group s_2. $(s_i = \{n, p, c\})$	Generated by **Table 9**

Further, $\phi_{P,pal}^{a,r}$ is the number of persons who are protected by monoclonal antibodies in age group a and clinical risk group r and $\phi_{P,vac}^{a,r}$ is the number of persons protected by vaccination in age group a and clinical risk group a at time t.

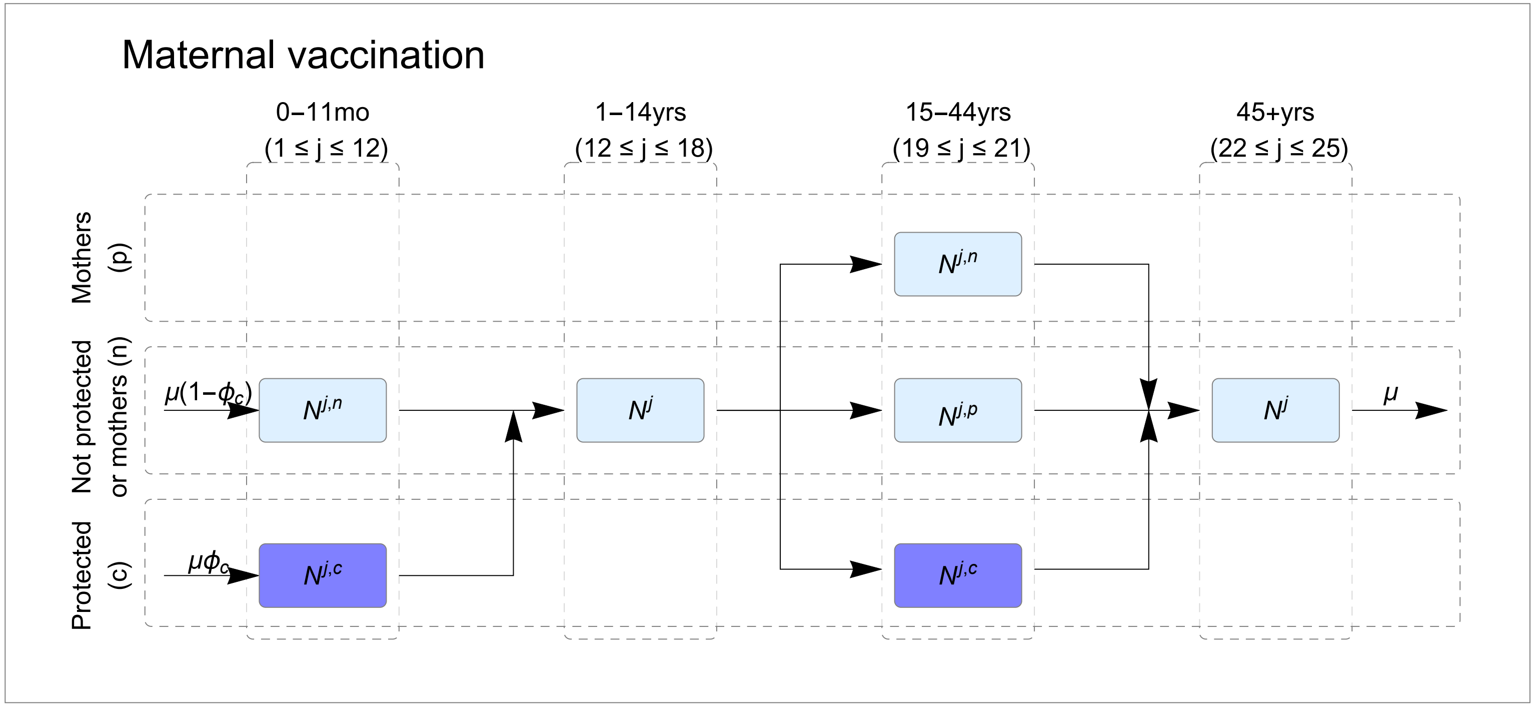

Figure 9: The relationship between maternal vaccine groups in the maternal vaccine intervention model. The parameters are the birth rate, μ, and the proportion of women who are newly mothers includedin the programme ϕ_c. The model ensure that all infants born to vaccinated women are protection (all into group M), otherwise they are born according to the dynamic maternal immunity assumption.

The initial conditions for this set of ODEs are given by **Equations (3)** with $N = N^{a,r,s}$ and $V_P^{a,r,s} = 0$.

5 Economic model

All of the parameters of the economic model are given in **Table 3** of the main text.

5.1 Estimating annual incidence of outcomes

We estimated the annual incidence of four different RSV-related outcomes (GP consultations, hospital bed days, symptomatic infections, hospitalisations and deaths) under the existing Palivizumab programme by synthesising recent incidence estimates for RSV outcomes in England. The incidence of GP consultations and deaths are age-dependent and estimated from three sources for each age category: 0–5 years of age,[27] 5–55 years[28] and for 55 years and over.[29] For hospital admissions and number of bed days, the incidence was dependent on age and clinical risk status. Reeves et al.[30] gives the estimated number of hospital bed days and hospital admissions for high-risk (HR) and not-at-risk (NR) infants up to 11 months of age. For the individuals aged 1-4 years and 5-14 years (which are NR), the number of hospital admissions is estimated from Reeves et al. 2017,[17] and Taylor et al[29] and the number of bed days per hospitalisation is 2 days.[31] For persons aged 15-64 years and 65+ years, we used Fleming et al.[28] and data from PHE and assuming the average number of bed days per hospitalisations is 3 days.[33] For all the studies highlighted above, the mean μ and 95% CI (c_l, c_u) are given, therefore, we fit a probability distribution using **Fitting procedure 2:**

Fitting procedure 2 *If CI are symmetric:* $(|(c_u - \mu)| = |(c_l - \mu)|)$*:* The fitted distribution is $\mathcal{N}(\mu, (c_u - \mu)/2)$. *If CI are non-symmetric:* $|(c_u - \mu)| \neq |(c_l - \mu)|$*:* By choosing the parametric distributions, $\mathcal{X} = \{Gamma(\alpha, \mu/\alpha), \mathcal{LN}(\log(u), \sigma), Weibull(a, \mu(\Gamma(1 + 1/a))^{-1})\}$, (chosen such that $\forall X \in \mathcal{X}$, $\mathbb{E}[X] = \mu$), the fitted parameters are found by solving the non-linear equation $\forall \theta \in \Theta = \{\alpha, \mu, a\}$

$$\int_{c_l}^{c_u} p_\theta(x)dx - 0.95 = 0 \tag{22}$$

to find the fitted values $\tilde{\Theta}$. We choose the uncertainty according to the distribution whose fitted parameter, $\tilde{\theta}$ minimising the cost function

$$\lambda(\tilde{\theta}) = (P_{\tilde{\theta}}(c_l) - 0.025)^2 + (P_{\tilde{\theta}}(c_u) - 0.975)^2 \tag{23}$$

A summary of age-specific annual incidence rates for GP consultations, hospital admissions, number of bed days and deaths is given in **Figure 10**. **Fitting procedure 2** is also used to estimate all the probability distributions in **Tables 2 and 3** of the main text.

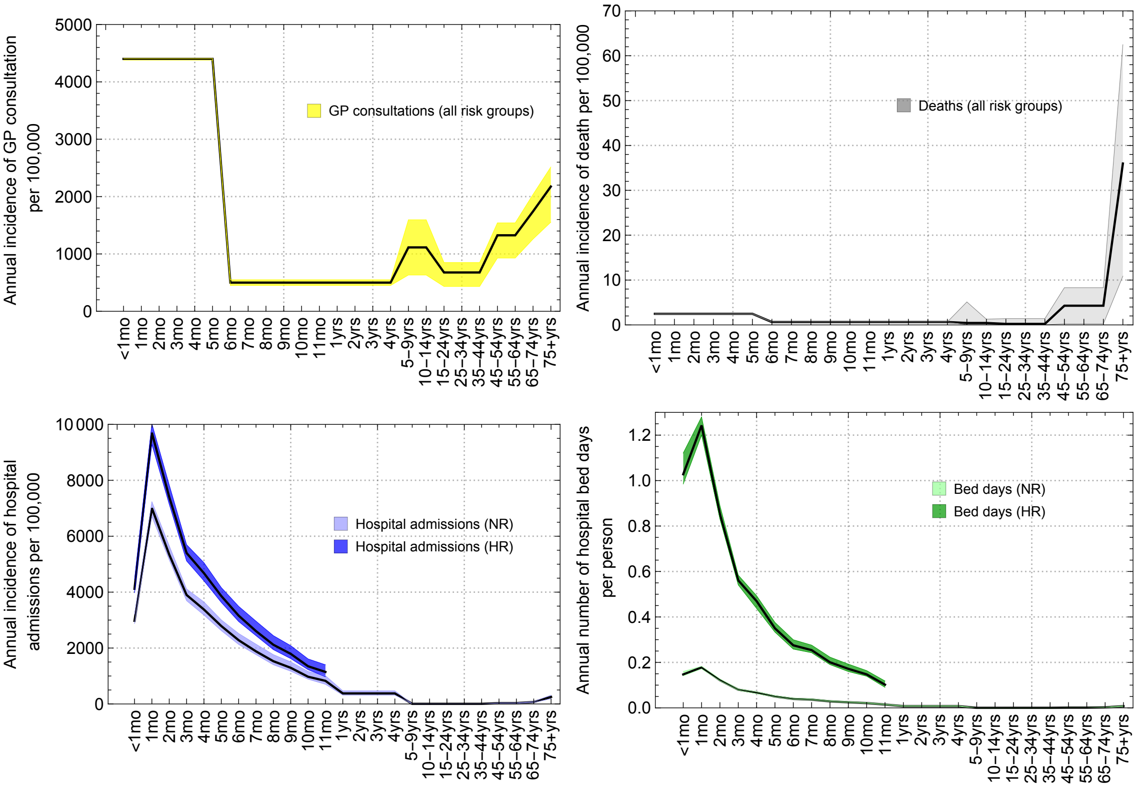

Figure 10: Estimated annual incidence of GP consultations (top left), deaths (top right), hospital admission (bottom left) and number of bed days (bottom right) per 100,000 persons in each age group (x-axis) and clinical risk group.

5.2 QALY loss due to death

Assuming an average life expectancy per person of 81.0 years,[34] then using SF-6D population norms with annual weighting of x_a per year,[35] the quality adjusted life expectancy is given by $\sum_{a=0}^{81} x_a = 65.94$. Given death occurs at year of life a_i, then the QALY loss, assuming a discounting rate of r = 0.035, is given by

$$\mathbb{E}[Q_D^{a_i}] = \sum_{a=a_i}^{81} x_a \exp(-0.035(a - a_i)) \tag{24}$$

We assume that the standard deviation of the life expectancy at age a_i is 10% of the current life expectancy ($Q_D^{a_i} \sim \mathcal{N}(\mathbb{E}[Q_D^{a_i}], 0.1(\mathbb{E}[Q_D^{a_i}]))$.

5.3 Cost-effectiveness

If X is the prophylactic associated with intervention programme P, then the cost of treatment (Θ^P), cost of administration (Δ^P) cost of purchasing (B^P) and the total QALY loss ($\mathcal{Q}^P$) associated with each treatment over the time horizon is given by:

$$\Theta^P = \sum_{w=1}^{52*T} Z_{P,t_w}^{a,r} (r_G^a \Theta_{GP} + r_B^{a,r} \Theta_H^a) e^{-rw/52} \tag{25}$$

$$\Delta^P = \Delta_X \sum_{w=1}^{52*T} D_{P,t_w}^{a,r} e^{-rw/52} \tag{26}$$

$$B^P = \rho_X \sum_{w=1}^{52*T} D_{P,t_w}^{a,r} e^{-rw/52} \tag{27}$$

$$\mathcal{Q}^P = \sum_{w=1}^{52*T} Z_{P,t_w}^{a,r} (r_S^a Q_G + r_H^{a,r} Q_H^a + r_D^{a,r} Q_D^a) e^{-rw/52} \tag{28}$$

The formula for the maximum price per dose to implement programme P in an existing programme C to remain cost-effective at an 20,000£/QALY threshold is given by:

$$\rho(P, C) = \frac{20000(\mathcal{Q}^C - \mathcal{Q}^P) - (\Theta^P - \Theta^C) - (\Delta^P - \Delta^C)}{\sum_{w=1}^{52*T} D_{P,t_w}^{a,r} e^{-rw/52} - \sum_{w=1}^{52*T} D_{C,t_w}^{a,r} e^{-rw/52}} \tag{29}$$

References

[1] Office for National Statistics. Births in England and Wales: 2017; 2018. Available from: https://www.ons.gov.uk/peoplepopulationandcommunity/birthsdeathsandmarriages/livebirths/bulletins/birthsummarytablesenglandandwales/2017.

[2] Stensballe LG, Ravn H, Kristensen K, Agerskov K, Meakins T, Aaby P, et al. Respiratory syncytial virus neutralizing antibodies in cord blood, respiratory syncytial virus hospitalization, and recurrent wheeze. J Allergy Clin Immun. 2009;123(2):398–403. Available from: http://dx.doi.org/10.1016/j.jaci.2008.10.043.

[3] Mossong J, Hens N, Jit M, Beutels P, Auranen K, Mikolajczyk R, et al. Social contacts and mixing patterns relevant to the spread of infectious diseases. PLoS Med. 2008;5(3):e74.

[4] van Hoek AJ, Andrews N, Campbell H, Amirthalingam G, Edmunds WJ, Miller E. The Social Life of Infants in the Context of Infectious Disease Transmission; Social Contacts and Mixing Patterns of the Very Young. PLoS ONE. 2013;8(10):1–7.

[5] Scott PD, Ochola R, Ngama M, Okiro EA, James Nokes D, Medley GF, et al. Molecular Analysis of Respiratory Syncytial Virus Reinfections in Infants from Coastal Kenya. The Journal of Infectious Diseases. 2006;193(1):59–67. Available from: https://academic.oup.com/jid/article-lookup/doi/10.1086/498246.

[6] Hall CB, Walsh EE, Long CE, Schnabel KC. Immunity to and frequency of reinfection with respiratory syncytial virus. J Infect Dis. 1991;163(4):693–698.

[7] Ogilvie MM, Vathenen AS, Radford M, Codd J, Key S. Maternal antibody and respiratroy syncytial virus infection in infancy. J Med Virol. 1981;7:263–271.

[8] Glezen WP, Paredes A, Allison JE, Taber LH, Frank AL. Risk of respiratory syncytial virus infection for infants from low-income families in relationship to age, sex, ethnic group, and maternal antibody level. J Pediatr. 1981;98(5):708–715.

[9] Ochola R, Sande C, Fegan G, Scott PD, Medley GF, Cane PA, et al. The level and duration of RSV-specific maternal IgG in infants in kilifi Kenya. PLoS ONE. 2009;4(12):4–9.

[10] DeVincenzo JP, Wilkinson T, Vaishnaw A, Cehelsky J, Meyers R, Nochur S, et al. Viral load drives disease in humans experimentally infected with respiratory syncytial virus. Am J Resp Crit Care. 2010;182(10):1305–1314.

[11] Okiro EA, White LJ, Ngama M, Cane PA, Medley GF, Nokes DJ. Duration of shedding of respiratory syncytial virus in a community study of Kenyan children. BMC Infectious Diseases. 2010;10:15.

[12] Henderson FW, Collier AM, Clyde WA, Denny FW. Respiratory-Sycytial-Virus infections, refinections and immunity: A prospective, longitudinal study in young children. New England Journal of Medicine. 1974;290(14).

[13] Watt PJ, Robinson BS, Pringle CR, Tyrrel DAJ. Determinants of susceptibility to challenge and the antibody response of adult volunteers given experimental respiratory syncytial virus vaccines. Vaccine. 1990;8(3):231–236.

[14] Munywoki PK, Koech DC, Agoti CN, Bett A, Cane PA, Medley GF, et al. Frequent Asymptomatic Respiratory Syncytial Virus Infections during an Epidemic in a Rural Kenyan Household Cohort. Journal of Infectious Diseases. 2015;212(10):1711–1718.

[15] Zhao H, Green H, Lackenby A, Donati M, Ellis J, Thompson C, et al. A new laboratory-based surveillance system (Respiratory Datamart System) for influenza and other respiratory viruses in England: Results and experience from 2009 to 2012. Eurosurveillance. 2014;19(3):1–10.

[16] Glezen WP, Taber LH, Frank AL, Kasel JA. Risk of primary infection and reinfection with respiratory syncytial virus. Am J Dis Child. 1986;140(6):543–6. Available from: http://www.ncbi.nlm.nih.gov/pubmed/3706232.

[17] Reeves RM, Hardelid P, Gilbert R, Warburton F, Ellis J, Pebody RG. Estimating the burden of respiratory syncytial virus (RSV) on respiratory hospital admissions in children less than five years of age in England, 2007-2012. Influenza Other Respi Viruses. 2017;11(3):122–129.

[18] La BB, Miasojedow Z, And EM, Vihola M. Adaptive Parallel Tempering Algorithm; 2012.

[19] Cunningham JP, Hennig P, Lacoste-Julien S. Gaussian Probabilities and Expectation Propagation. 2011;2:1–56. Available from: http://arxiv.org/abs/1111.6832.

[20] NHS. Clinical Commissioning Policy: Palivizumab To Reduce The Risk Of RSV In High Risk Infants NHS Commissioning Board Clinical Commissioning Policy: Palivizumab To Reduce The Risk Of Respiratory Syncytial Virus (RSV) In High Risk Infants; 2012. Available from: https://www.engage.england.nhs.uk/consultation/ssc-area-e/supporting{_}documents/e8apolicy.pdf.

[21] Steurer MA, Baer RJ, Keller RL, Oltman S, Chambers CD, Norton ME, et al. Gestational Age and Outcomes in Critical Congenital Heart Disease. Pediatrics. 2017 oct;140(4):e20170999. Available from: http://www.ncbi.nlm.nih.gov/pubmed/28885171.

[22] Ambrose CS, Jiang X, Mavunda K. 737. The Prevalence of Diagnosed Chronic Lung Disease in US Infants by Gestational Age: Implications for RSV Policy. Open Forum Infectious Diseases. 2018;5(suppl_1):S264–S265.

[23] Green CA, Yeates D, Goldacre A, Sande C, Parslow RC, McShane P, et al. Admission to hospital for bronchiolitis in England: trends over five decades, geographical variation and association with perinatal characteristics and subsequent asthma. Archives of disease in childhood. 2016 feb;101(2):140–6. Available from: http://www.ncbi.nlm.nih.gov/pubmed/26342094http://www.pubmedcentral.nih.gov/articlerender.fcgi?artid=PMC4752648.

[24] Baguelin M, Flasche S, Camacho A, Demiris N, Miller E, Edmunds WJ. Assessing optimal target populations for influenza vaccination programmes: an evidence synthesis and modelling study. PLoS Med. 2013;10(10).

[25] Office for National Statistics. Births by parents' characteristics: 2017; 2019. Available from: https://www.ons.gov.uk/peoplepopulationandcommunity/birthsdeathsandmarriages/livebirths/datasets/birthsbyparentscharacteristics.

[26] Atkins KE, Fitzpatrick MC, Galvani AP, Townsend JP. Cost-Effectiveness of Pertussis Vaccination During Pregnancy in the United States. American Journal of Epidemiology. 2016 jun;183(12):1159–1170. Available from: https://academic.oup.com/aje/article-lookup/doi/10.1093/aje/kwv347.

[27] Cromer D, van Hoek AJ, Newall AT, Pollard AJ, Jit M. Burden of paediatric respiratory syncytial virus disease and potential effect of different immunisation strategies: a modelling and cost-effectiveness analysis for England. The Lancet Public Health. 2017 aug;2(8):e367–e374. Available from: https://www.sciencedirect.com/science/article/pii/S2468266717301032.

[28] Fleming DM, Taylor RJ, Lustig RL, Schuck-Paim C, Haguinet F, Webb DJ, et al. Modelling estimates of the burden of Respiratory Syncytial virus infection in adults and the elderly in the United Kingdom. BMC Infec Dis. 2015;15(1):443. Available from: http://www.biomedcentral.com/1471-2334/15/443http://www.pubmedcentral.nih.gov/articlerender.fcgi?artid=4618996{\&}tool=pmcentrez{\&}rendertype=abstract.

[29] Taylor S, Taylor R, Lustig R, Schuck-Paim C, Haguinet F, Webb D, et al. Modelling estimates of the burden of respiratory syncytial virus infection in children in the UK. BMJ Open. 2016;6:e009337. Available from: http://www.pubmedcentral.nih.gov/articlerender.fcgi?artid=4618996{\&}tool=pmcentrez{\&}rendertype=abstract.

[30] Reeves RM, Hardelid P, Panagiotopoulos N, Minaji M, Warburton F, Pebody R. Burden of hospital admissions caused by respiratory syncytial virus (RSV) in infants in England: a data linkage modelling study. Journal of Infection. 2019 feb;Available from: https://linkinghub.elsevier.com/retrieve/pii/S0163445319300659.

[31] Hardelid P, Verfuerden M, McMenamin J, Smyth R, Gilbert R. The contribution of child, family and health service factors to respiratory syncytial virus (RSV) hospital admissions in the first 3 years of life: birth cohort study in Scotland, 2009 to 2015. Eurosurveillance. 2019;Available from: www.eurosurveillance.org.

[32] A Study to Evaluate the Safety of MEDI8897 for the Prevention of Medically Attended Respiratory Syncytial Virus(RSV) Lower Respiratory Track Infection (LRTI) in High-risk Children;. Available from: https://clinicaltrials.gov/ct2/show/NCT03959488?term=MEDI8897{\&}rank=1.

[33] Widmer K, Zhu Y, Williams JV, Griffin MR, Edwards KM, Talbot HK. Rates of hospitalizations for respiratory syncytial virus, human metapneumovirus, and influenza virus in older adults. The Journal of infectious diseases. 2012 jul;206(1):56–62. Available from: http://www.ncbi.nlm.nih.gov/pubmed/22529314http://www.pubmedcentral.nih.gov/articlerender.fcgi?artid=PMC3415933.

[34] ONS. National life tables, UK - Office for National Statistics; 2018. Available from: https://www.ons.gov.uk/peoplepopulationandcommunity/birthsdeathsandmarriages/lifeexpectancies/bulletins/nationallifetablesunitedkingdom/2015to2017.

[35] Bernard B. SF-6D Population Norms. 2012;.
