# Supplementary material for: Evaluating the next generation of RSV intervention strategies: a mathematical modelling study and cost-effectiveness analysis
Source: BMC Med. 2020 Nov 18;18:348. doi: 10.1186/s12916-020-01802-8 (PMC7672821; doi:10.1186/s12916-020-01802-8)
Supplement: Supplementary file 2 — Additional file 2: Figure S1. DIC for the ten model choices. Figure S2. Top. The proportion of infants born with protection over an epidemic season Figure S2. Bottom. A comparison of the posterior distributions for the detection model. Figure S3. A comparison between the model-predicted number of detected samples and the annual number of positive samples from RDMS. Figure S4. Top. The source of the infection for each demographic group in the analysis. Figure S4. Bottom. The magnitude of the force of infection relative to age group 1. Figure S5. Smooth histogram plots comparing the prior and posterior distributions. Figure S6. Estimated per-infection probability of GP consultations (top left), deaths (top right), hospital admission (bottom left) and number of bed days (bottom right). Figure S7. The total discount QALY loss over ten years. Figure S8. Impact of intervention programmes at preventing total proportion of RSV-related deaths. [file 12916_2020_1802_MOESM2_ESM.pdf]

# Additional file 2 for Evaluating the next generation of RSV intervention strategies: A mathematical modelling study and cost-effectiveness analysis
# Results

by David Hodgson, Richard Pebody, Jasmina Panovska-Griffiths, Marc Baguelin, and Katherine Atkins

## Contents

**1 Model choice** **2**

**2 RSV Epidemiology** **4**
2.1 Estimated incidence . . . . . . . . . . 4
2.2 Posterior distributions of epidemic parameters . . . . . . . . . . 6

**3 Probability of clinical outcomes** **7**

**4 Impact of intervention programmes** **8**
4.1 Optimal period of administration . . . . . . . . . . 8
4.2 Outcomes averted . . . . . . . . . . 9

# 1 Model choice

Calculating the DIC for the 10 proposed model choices (two maternal immunity and five detection models) suggests that dynamic maternal immunity with an exponential detection model best fits the data (**Figure 1**). For all five of the detection models considered, the dynamic immunity model better fitted the data than the static immunity model. The corresponding values for $p_R$ and the detection probability is given in (**Figure 2**). The results which follow this section all refer to this model choice.

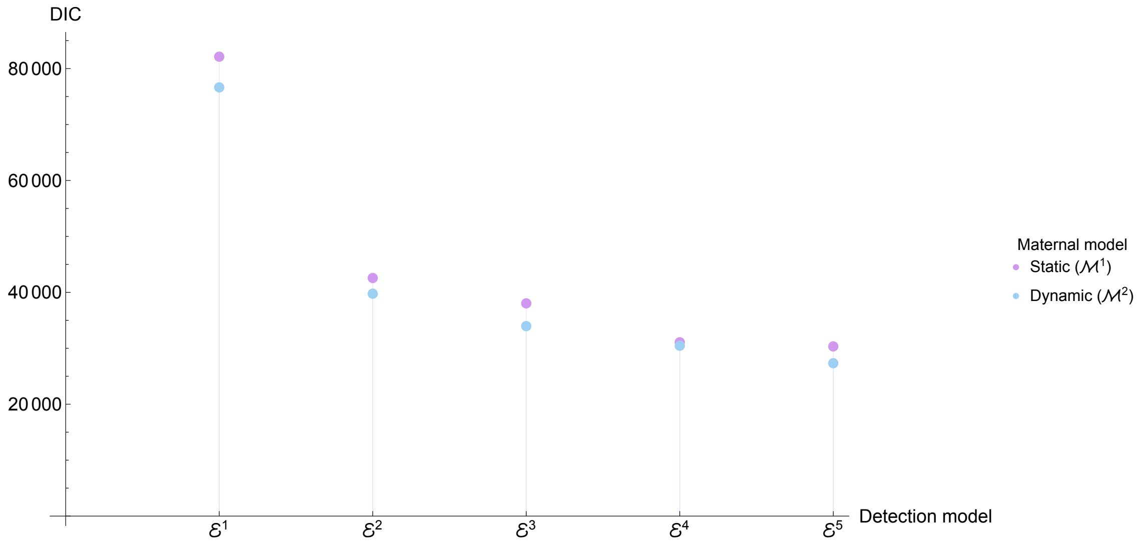


**Figure 1:** DIC for the 5 detection model structures, and the 2 maternal immunity model structures. Best fitting model is the exponential detection model ($\mathcal{E}^5$) with the dynamic maternal immunity model ($\mathcal{M}^2$).

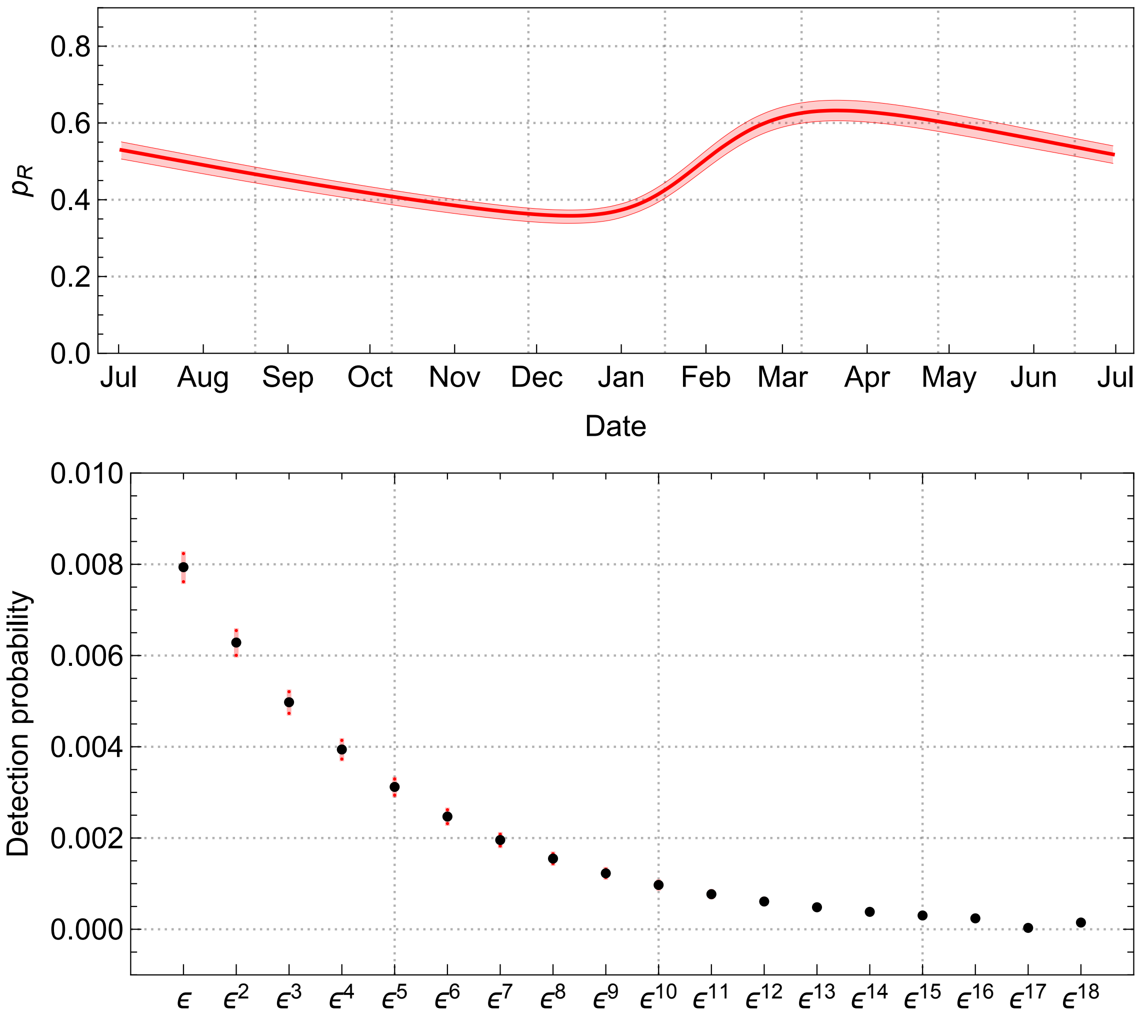


**Figure 2:** Top: The proportion of infants born with protection ($p_R$) over an epidemic season for the dynamic maternal immunity model. Bottom: A comparison of the posterior distributions for the detection model, $\mathcal{E}^5$, where black points indicates the mean values and the red points indicated the lower and upper credible intervals

# 2 RSV Epidemiology

## 2.1 Estimated incidence

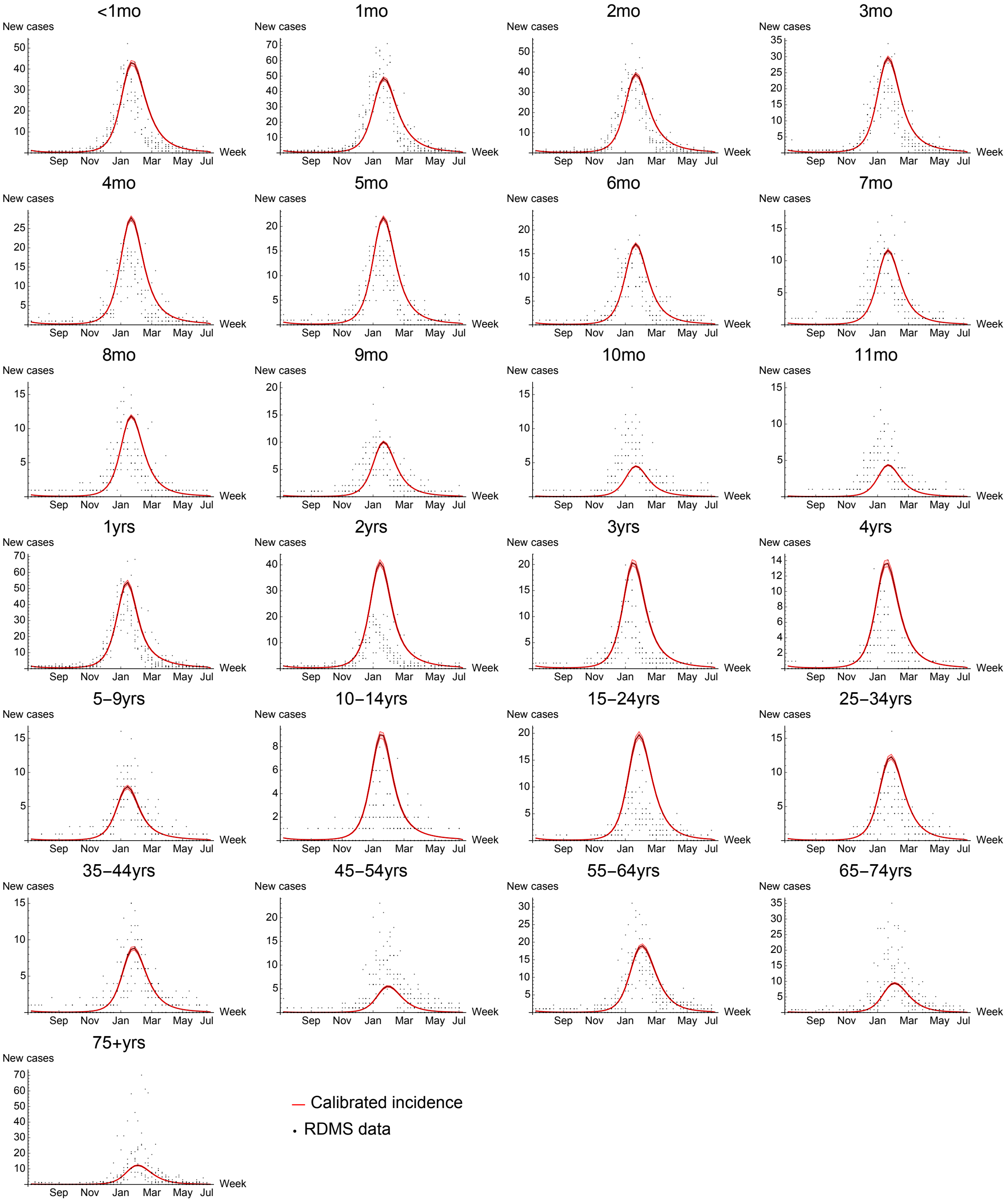


**Figure 3:** A comparison between the model-predicted number of detected samples during week $t$, ($Z^a_{w_t}\epsilon^a$, red line), estimated from averaging 1,000 samples from the posterior distribution during the third year of simulation, and the annual number of positive samples from RDMS ($d^a_{w_t}$, black dots) for age group $a$.

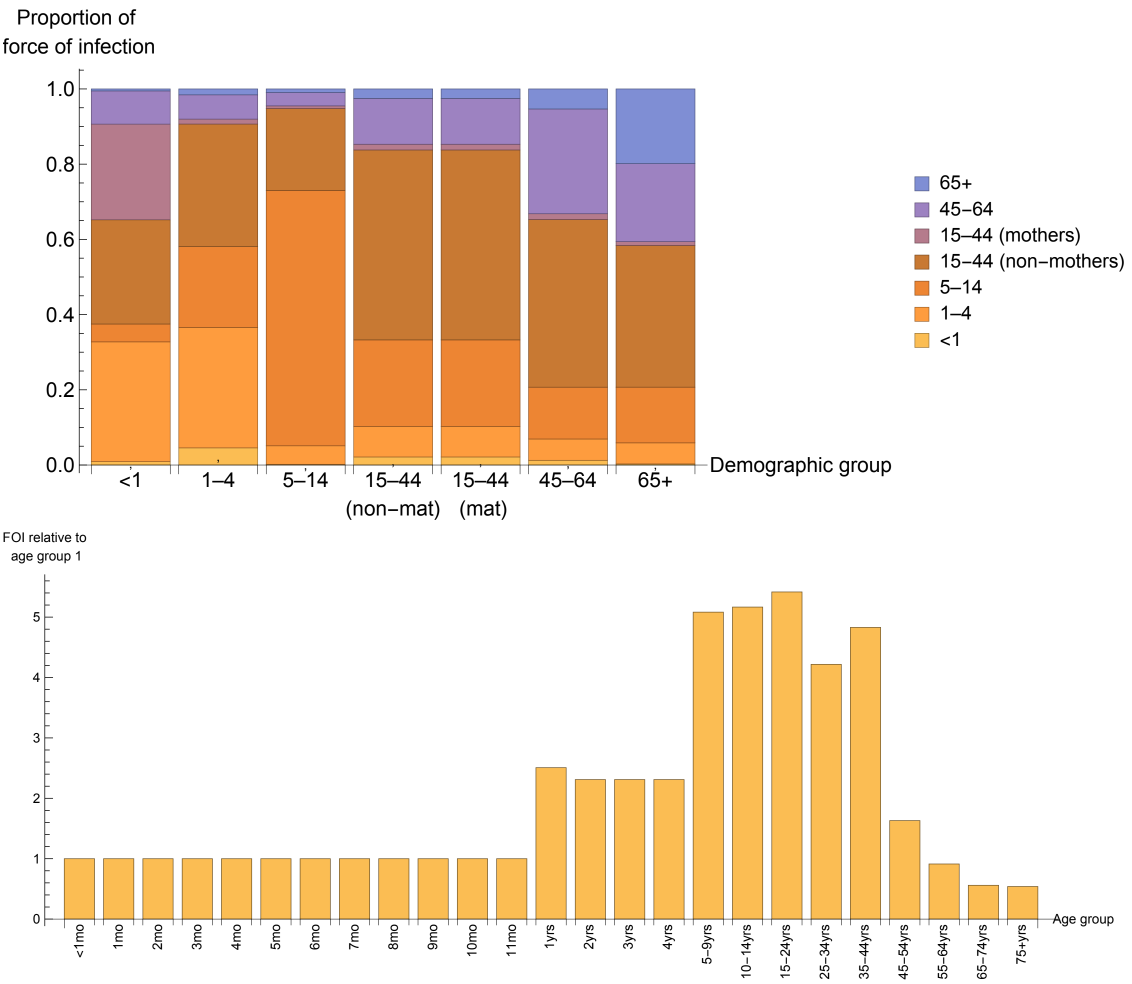


**Figure 4:** Top: the source of the infection for each demographic group in the analysis. Bottom: The magnitude of the force of infection relative to age group 1. The force of infection for both Figures was estimated using the third year of simulation

## 2.2 Posterior distributions of epidemic parameters

Model parmaeters ($\mathcal{M}^2$, $\mathcal{E}^5$)

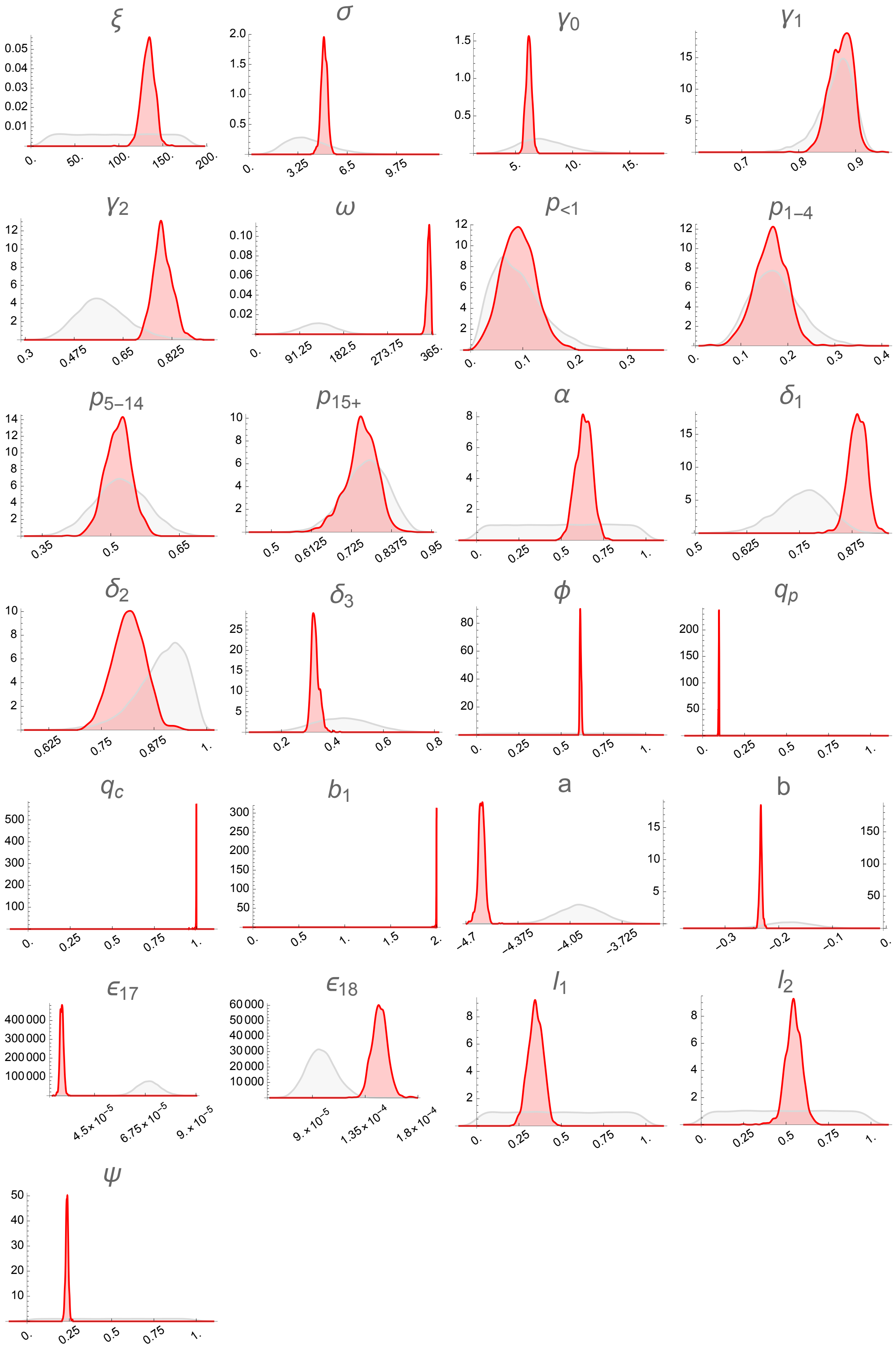


**Figure 5:** Smooth histogram plots comparing the the prior (gray) and posterior (red) distributions for each of the inferred parameters

# 3 Probability of clinical outcomes

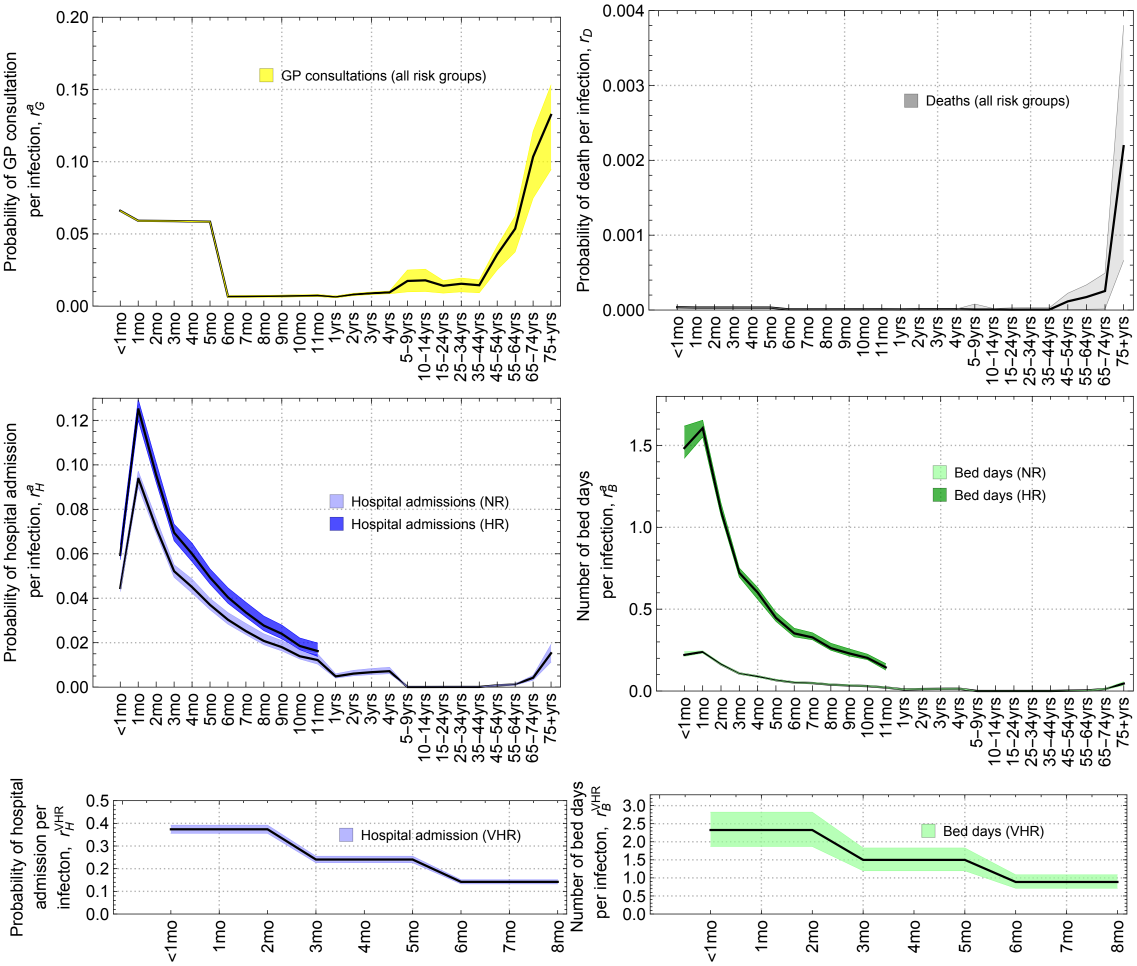


**Figure 6:** Estimated per-infection probability of GP consultations (top left), deaths (top right), hospital admission (bottom left) and number of bed days (bottom right) in each age group (x-axis) and clinical risk group.

# 4 Impact of intervention programmes

## 4.1 Optimal period of administration

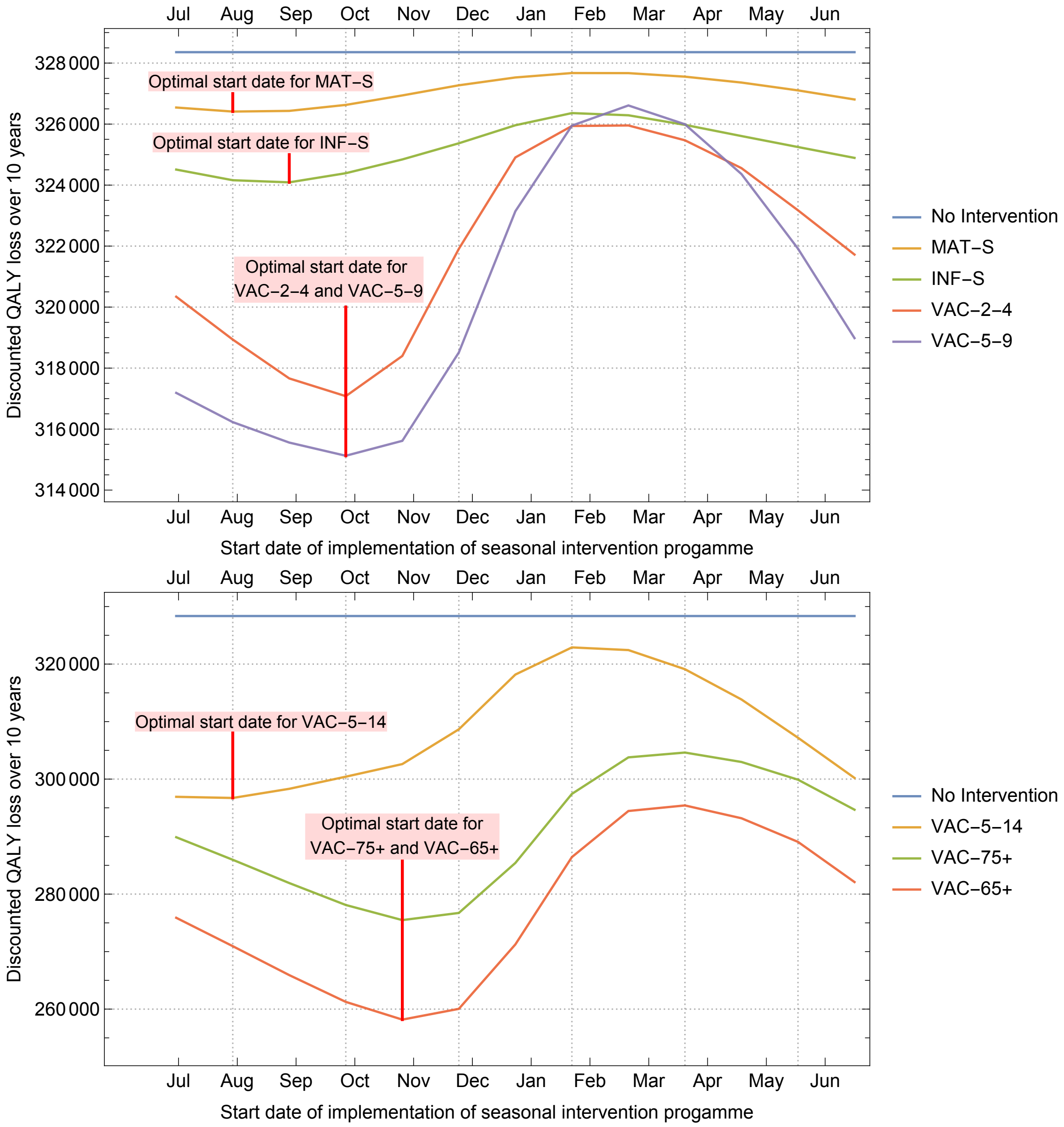


**Figure 7:** The total discount QALY loss over ten years when a seasonal programme starts administration on the month given on the x-axis.

## 4.2 Outcomes averted

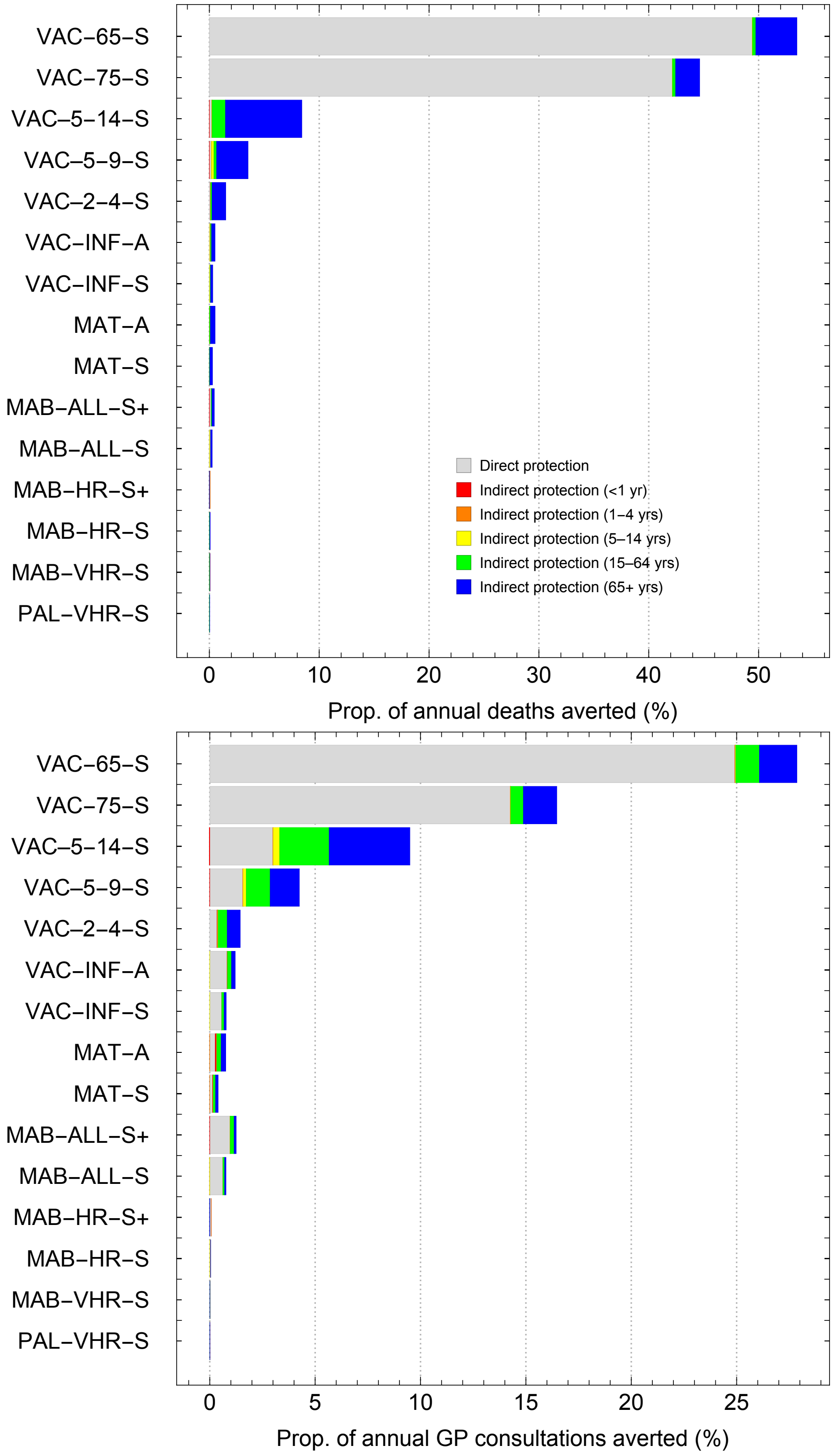


**Figure 8:** Impact of intervention programmes at preventing total proportion of RSV-related deaths. Gray segments of bars show direct protection and coloured segments of bars indicate indirect protection.
